# Synthesis, biological activity and molecular modelling studies of shikimic acid derivatives as inhibitors of the shikimate dehydrogenase enzyme of *Escherichia coli*

**DOI:** 10.1080/14756366.2017.1422125

**Published:** 2018-01-24

**Authors:** Dulce Catalina Díaz-Quiroz, César Salvador Cardona-Félix, José Luis Viveros-Ceballos, Miguel Angel Reyes-González, Franciso Bolívar, Mario Ordoñez, Adelfo Escalante

**Affiliations:** a Departamento de Ingeniería Celular y Biocatálisis, Instituto de Biotecnología, Universidad Nacional Autónoma de México, Cuernavaca, México;; b CONACyT – Instituto Politécnico Nacional, Centro Interdisciplinario de Ciencias Marinas, La Paz, México;; c Centro de Investigaciones Químicas, Universidad Autónoma del Estado de Morelos, Cuernavaca, México

**Keywords:** Shikimate dehydrogenase, enzyme inhibition, *Escherichia coli*, shikimic acid, amide derivative synthesis, molecular docking

## Abstract

Shikimic acid (SA) pathway is the common route used by bacteria, plants, fungi, algae, and certain Apicomplexa parasites for the biosynthesis of aromatic amino acids and other secondary metabolites. As this essential pathway is absent in mammals designing inhibitors against implied enzymes may lead to the development of antimicrobial and herbicidal agents harmless to humans. Shikimate dehydrogenase (SDH) is the fourth enzyme of the SA pathway. In this contribution, a series of SA amide derivatives were synthesised and evaluated for *in vitro* SDH inhibition and antibacterial activity against *Escherichia coli.* All tested compounds showed to be mixed type inhibitors; diamide derivatives displayed more inhibitory activity than synthesised monoamides. Among the evaluated compounds, molecules called **4a** and **4b** were the most active derivatives with IC_50_ 588 and 589 µM, respectively. Molecular modelling studies suggested two different binding modes of monoamide and diamide derivatives to the SDH enzyme of *E. coli*.

## Introduction

The shikimic acid (SA) pathway is the common route for aromatic amino acid biosynthesis existing in bacteria, fungi, yeasts, plants, algae and certain Apicomplexa parasites but it is absent in mammals^1^. This pathway is essential as is involved in the generation of structural blocks for protein synthesis, vitamins and electron-carrier compounds such as cofactors and quinones^2–4^. In recent years, enzymes of the SA pathway have been recognised as new prominent targets for enzyme inhibition discover because of their roles in microbial cell survival, understood biochemistry and presence in the clinically relevant spectrum of species^5^. In this sense, much effort has been made to the design of molecules that might act as antibacterial, fungicidal, antiparasitic and herbicidal agents harmless to humans.

Targeting the SA pathway has led to drug development of antibacterial agents for critical pathogenic bacteria such as the tuberculosis responsible agent *Mycobacterium tuberculosis*^6^ and *Helicobacter pylori*^7^ a primary causative factor for gastrointestinal illnesses. The anti-parasitic against the malaria parasite *Plasmodium falciparum*^8^ and toxoplasmosis parasite *Toxoplasma gondhii*^9^, as well as one of the most widely used herbicide during the past three decades glyphosate [N-(phosphonomethyl) glycine] the active ingredient in RoundUp^®^, Zero^®^ and Tumbleweed^®10^,^^11^^.

Shikimate dehydrogenase (SDH, E.C. 1.1.1.25), the fourth enzyme in the SA pathway catalyses the reversible reduction of 3-dehydroshikimate (DHS) to SA using NADPH as co-substrate. SDH is a member of an oxide-reductase family whose crystallographic structure in several organisms have been determined either in the apoenzyme form or binary and ternary complexes^12^. The shared three-dimensional fold consists of two α/β domains linked by a pair of α-helices, the substrate-binding site is mainly delineated by residues from the N-terminal domain whereas the cofactor binding site is comprising in the C-terminal domain that adopts a Rossmann fold. Functional and structural studies on SDH enzymes opened a platform for the development of SDH inhibitors as few reports exist compared to other enzymes of the SA pathway.

First attempts to identify SDH inhibitors were based on the elucidation of interaction requirements for substrate binding to the active site of the enzyme. To determine the binding mode of SA and DHS to the *Pisum sativum* SDH enzyme, Balinsky and Davies analysed several substrate similar compounds for competitive inhibition^13^. Both a carbonyl group in the C1 position and a hydroxyl group corresponding to the 4-OH position were identified as significant determinants for ligand binding. The aldehyde vanillin proved to be a potent competitive inhibitor (*K_i_*, SA = 93 µM) by the observations of Baillie et al. for the non-specific nature of the C-1 carbonyl group in inhibitors which they tested as alkyl and aryl esters^14^.

High throughput screening provides a non-rational approach for the identification of new chemical scaffolds with inhibitory activity. Using a library of 5000 compounds Han et al. identified five novel SDH inhibitors of *H. pylori*. Interestingly curcumin showed to be a non-competitive inhibitor (*K_i_*, SA = 6 µM) with moderate *in vitro* antibacterial activity (MIC = 16 µg/ml)^15^. By employing a similar screening strategy, other phenolic compounds as epigallocatechin gallate were identified as inhibitors of SDH of *Pseudomonas putida* (IC_50_ = 3 µM)^16^. Most recently, virtual screening and site-moiety maps that rely on binding environment modelling led to the identification of SDH inhibitors with IC_50_ values around 100 µM (competitive)^17^ and 2 µM (non-specified)^18^.

SA has probed to inhibit SDH enzyme of *Escherichia coli* (EcSDH) (*K_i_* = 160 µM)^19^. Taking into account the previous observation that inhibitors carbonyl C-1 group can correspond to that of an aldehyde, ester or carboxylic acid, in this contribution we explored the carbonyl nature by synthesising amides harbouring compounds to test new derivatives for SDH inhibitor discovery. Based on this hypothesis, an amide group was introduced to evaluate the bioisosteric replacement in which the carboxylate moiety of SA that harbours hydrogen bonds accepting groups was changed to the amide that can either donate and accept hydrogen bonds. We also assessed the possible inhibitory activity of SA derivative amides by hydroxyl ended linear alkyl chains of different lengths used as N-substituents. As a continuation, diamides composed of two SA molecules were synthesised based on the structure of vanillin and curcumin in which two nuclei of an identified inhibitor are linked by an aliphatic chain leading to inhibition enhancement. Enzyme kinetic studies for inhibition-type identification and antibacterial activity were also assessed. Finally, molecular docking was performed for an understanding of structure-activity relationships.

## Experimental

### Chemistry

#### Materials and methods

All commercial materials were used as received unless otherwise noted. Flash chromatography was performed on 230–400 mesh Silica Flash 60^®^. Thin layer chromatography was performed on pre-coated TLC sheets of silica gel (60 F_254_, Merck) and the plates were visualised by UV-light and iodine vapours or vanillin-sulphuric acid followed by heating. Melting points were determined with a Fisher–Johns apparatus and are uncorrected. ^1^H and ^13^C NMR spectra were recorded on a Varian instrument (400 MHz for ^1^H) using the TMS and the residual solvent signal as internal standards; chemical shifts (*δ*) are expressed in parts per million (ppm) and coupling constants (*J*) in Hertz. High-resolution FAB^+^ mass spectra (HRMS) were obtained on a JEOL MStation MS-700.

#### (3R,4S,5R)-3,4,5-tri[(tert-butyldimethylsilyl)oxy]cyclohex-1-enecarboxylic acid (2)

To a solution of (-)-shikimic acid **1** (1.0 g, 5.74 mmol) in anhydrous dimethylformamide (DMF) (12 ml) was added imidazole (4.4 g, 64.5 mmol) and *tert*-butyldimethylsilyl chloride (8.7 g, 57.4 mmol). The reaction mixture was stirred at 70 °C for 20 h under nitrogen atmosphere. Then 10 ml of H_2_O were added and the reaction mixture was extracted with CH_2_Cl_2_ (3 × 15 ml). The combined organic layers were dried over Na_2_SO_4_, filtered and concentrated under reduced pressure. The crude product was purified by column chromatography using Hex:AcOEt (92:8) as eluent, obtaining (2.52 g, 85% yield) of **2** as a white solid. M.p. 169–172 °C. IR (neat): ṽ = 2929, 1690, 1256 cm^−1^. [α]_D_ = –85.7 (*c* 1.0, CH_2_Cl_2_). ^1^H NMR (400 MHz, CDCl_3_): *δ* = 0.07 (s, 12H, CH_3_Si), 0.11 (s, 3H, CH_3_Si), 0.12 (s, 3H, CH_3_Si), 0.84 (s, 9H, (CH_3_)_3_CSi), 0.86 (s, 9H, (CH_3_)_3_CSi), 0.94 (s, 9H, (CH_3_)_3_CSi), 2.17 (dd, *J* = 17.8, 1.0 Hz, 1H, H-6), 2.58 (ddt, *J* = 17.8, 3.0, 3.0 Hz, 1H, H-6′), 3.74–3.78 (m, 1H, H-4), 3.98–4.02 (m, 1H, H-5), 4.62–4.66 (m, 1H, H-3), 6.82–6.85 (m, 1H, H-2) ppm. ^13^C NMR (100 MHz, CDCl_3_): *δ* = −4.7 (CH_3_Si), −4.6 (CH_3_Si), −4.5 (CH_3_Si), −4.2 (CH_3_Si), 18.1 (*C*(CH_3_)_3_), 18.3 (*C*(CH_3_)_3_), 18.7 (*C*(CH_3_)_3_), 25.9 (C(*C*H_3_)_3_), 26.0 (C(*C*H_3_)_3_), 26.4 (C(*C*H_3_)_3_), 29.4 (C-6), 68.4 (C-4), 70.0 (C-3), 72.6 (C-5), 126.7 (C-1), 143.3 (C-2), 172.9 (C=O) ppm. HRMS (FAB^+^): calcd. for C_25_H_53_O_5_Si_3_ [M + H]^+^, *m/z* 517.3201; found for [M + H]^+^, *m/z* 517.4024.

#### Synthesis of monoamides (3a–3d)

To a solution of **2** (250 mg, 0.5 mmol) in dry CH_2_Cl_2_ (6 ml), oxalyl chloride (1.2 eq) and one drop of DMF were added slowly, and the reaction was stirred at room temperature for 3.0 h under nitrogen atmosphere. Then, the solvent was evaporated under reduced pressure, and the formed acid chloride was slowly added to a solution containing the appropriate hydroxylamine (10 eq) and *N*, *N*-diisopropylethylamine (DIPEA) (2 eq) in dry CH_2_Cl_2_ (6 ml). The reaction mixture was stirred at room temperature for 3 h under nitrogen atmosphere. After completion of the reaction, H_2_O (20 ml) was added and extracted with AcOEt (30 ml). The organic layer was washed with a saturated aqueous NaHCO_3_ solution (3 × 20 ml), dried over Na_2_SO_4_, filtered and concentrated under reduced pressure. Tetrabutylammonium fluoride (TBAF) (1.0 M in tetrahydrofuran (THF), 5 eq) was added dropwise to a solution of this crude in THF (3 ml) at 0 °C and the reaction mixture was stirred at room temperature for 12 h. The reaction mixture was passed through Dowex^®^ 50WX8 (H^+^) column and eluted with H_2_O. The collected fractions were combined, concentrated and subjected to flash chromatography using AcOEt:MeOH (90:10) as eluent, obtaining the titled compounds **3a**–**3d** as clear oils.

#### (3*R*,4*S*,5*R*)-3,4,5-trihydroxy-*N*-(3-hydroxypropyl)cyclohex-1-ene-1-carboxamide (3a)

70 mg, 63% yield. IR (neat): ṽ = 3303, 2931, 1661, 1606, 1061 cm^−1^. [α]_D_ = −84.9 (*c* 1.0, CH_3_OH). ^1^H NMR (400 MHz, CD_3_OD): *δ* = 1.74 (quint, *J* = 6.6 Hz, 2H, CH_2_), 2.15 (dd, *J* = 17.8, 6.2 Hz, 1H, H-6), 2.72 (dd, *J* = 17.8, 5.0 Hz, 1H, H-6′), 3.33 (t, *J* = 6.9 Hz, 2H, CH_2_N), 3.59 (t, *J* = 6.3 Hz, 2H, CH_2_O), 3.64 (dd, *J* = 7.8, 4.2 Hz, 1H, H-4), 3.98 (ddd, *J* = 7.4, 5.8, 5.8 Hz, 1H, H-5), 4.34 (dd, *J* = 3.9, 3.9 Hz, 1H, H-3), 6.39–6.42 (m, 1H, H-2) ppm. ^13^C NMR (100 MHz, CD_3_OD): *δ* = 32.3 (C-6), 33.3 (CH_2_), 38.0 (CH_2_N), 61.0 (CH_2_O), 68.0 (C-4), 68.4 (C-5), 73.2 (C-3), 133.0 (C-2), 134.3 (C-1), 171.0 (C=O) ppm. HRMS (FAB^+^): calcd. for C_10_H_18_NO_5_ [M + H]^+^, *m/z* 232.1185; found for [M + H]^+^, *m/z* 232.1239.

#### (3*R*,4*S*,5*R*)-3,4,5-trihydroxy-*N*-(4-hydroxybutyl)cyclohex-1-ene-1-carboxamide (3b)

80 mg, 69% yield. IR (neat): ṽ = 3321, 2929, 1662, 1607, 1060 cm^−1^. [α]_D_ = −87.6 (*c* 1.0, CH_3_OH). ^1^H NMR (400 MHz, CD_3_OD): *δ* = 1.50–1.64 (m, 4H, CH_2_), 2.15 (dd, *J* = 17.8, 6.2 Hz, 1H, H-6), 2.73 (dd, *J* = 17.8, 5.1 Hz, 1H, H-6′), 3.25 (t, *J* = 6.7 Hz, 2H, CH_2_N), 3.56 (t, *J* = 5.8 Hz, 2H, CH_2_O), 3.63 (dd, *J* = 7.8, 4.2 Hz, 1H, H-4), 3.98 (ddd, *J* = 7.7, 5.9, 5.9 Hz, 1H, H-5), 4.34 (dd, *J* = 4.0, 4.0 Hz, 1H, H-3), 6.38–6.41 (m, 1H, H-2) ppm. ^13^C NMR (100 MHz, CD_3_OD): *δ* = 27.1 (CH_2_), 31.1 (C6), 32.4 (CH_2_), 40.6 (CH_2_N), 62.7 (CH_2_O), 67.5 (C-4), 68.4 (C-5), 73.3 (C-3), 132.6 (C-2), 134.4 (C-1), 170.5 (C=O) ppm. HRMS (FAB^+^): calcd. for C_11_H_20_NO_5_ [M + H]^+^, *m/z* 246.1341; found for [M + H]^+^, *m/z* 246.1334.

#### (3*R*,4*S*,5*R*)-3,4,5-trihydroxy-*N*-(5-hydroxypentyl)cyclohex-1-ene-1-carboxamide (3c)

90 mg, 70% yield. IR (neat): ṽ = 3343, 2949, 1674, 1015 cm^−1^. [α]_D_ = −90.1 (*c* 1.0, CH_3_OH). ^1^H NMR (400 MHz, CD_3_OD): *δ* = 1.34–1.43 (m, 2H, CH_2_), 1.51–1.61 (quin, *J* = 6.9 Hz, 4H, CH_2_), 2.15 (dd, *J* = 17.7, 6.1 Hz, 1H, H-6), 2.73 (dd, *J* = 17.7, 5.0 Hz, 1H, H-6´), 3.24 (t, *J* = 7.0 Hz, 2H, CH_2_N), 3.55 (t, *J* = 6.5 Hz, 2H, CH_2_O), 3.63 (dd, *J* = 7.8, 4.1 Hz, 1H, H-4), 3.98 (ddd, *J* = 7.1, 5.8, 5.8 Hz, 1H, H-5), 4.34 (dd, *J* = 4.0, 4.0 Hz, 1H, H-3), 6.36–6.43 (m, 1H, H-2) ppm. ^13^C NMR (100 MHz, CD_3_OD): *δ* = 24.4 (CH_2_), 30.4 (C6), 32.3 (CH_2_), 33.4 (CH_2_), 40.7 (CH_2_N), 63.0 (CH_2_O), 68.0 (C-4), 68.4 (C-5), 73.2 (C-3), 132.6 (C-2), 134.4 (C-1), 170.5 (C=O) ppm. HRMS (FAB^+^): calcd. for C_12_H_22_NO_5_ [M + H]^+^, *m/z* 260.1498; found for [M + H]^+^, *m/z* 260.1490.

#### (3*R*,4*S*,5*R*)-3,4,5-trihydroxy-*N*-(6-hydroxyhexyl)cyclohex-1-ene-1-carboxamide (3d)

80 mg, 60% yield. IR (neat): ṽ = 3303, 2931, 1661, 1606, 1061 cm^−1^. [α]_D_ = −92.3 (*c* 1.0, CH_3_OH). ^1^H NMR (400 MHz, CD_3_OD): *δ* = 1.32–1.42 (m, 4H, CH_2_), 1.49–1.59 (m, 4H, CH_2_), 2.15 (dd, *J* = 17.8, 6.2 Hz, 1H, H-6), 2.73 (dd, *J* = 17.8, 5.1 Hz, 1H, H-6´), 3.23 (t, *J* = 7.0 Hz, 2H, CH_2_N), 3.54 (t, *J* = 6.5 Hz, 2H, CH_2_O), 3.63 (dd, *J* = 7.8, 4.2 Hz, 1H, H-4), 3.95–4.01 (m, 1H, H-5), 4.34 (dd, *J* = 4.0, 4.0 Hz, 1H, H-3), 6.37–6.40 (m, 1H, H-2) ppm. ^13^C NMR (100 MHz, CD_3_OD): *δ* = 27.0 (CH_2_), 28.0 (CH_2_), 31.0 (C6), 32.3 (CH_2_), 34.0 (CH_2_), 41.0 (CH_2_N), 63.0 (CH_2_O), 68.0 (C-4), 68.4 (C-5), 73.2 (C-3), 133.0 (C-2), 134.4 (C-1), 171.0 (C=O) ppm. HRMS (FAB^+^): calcd. for C_13_H_24_NO_5_ [M + H]^+^, *m/z* 274.1654; found for [M + H]^+^, *m/z* 274.1645.

#### Synthesis of diamides (4a–4c)

To a solution of **2** (0.25 g, 0.48 mmol) in dry CH_2_Cl_2_ (6 ml), oxalyl chloride (1.2 eq) and one drop of DMF were added slowly, and the reaction was stirred at room temperature for 3.0 h under nitrogen atmosphere. Then the solvent was evaporated under reduced pressure, and the formed acid chloride was slowly added to a solution containing the appropriate diamine (5 eq), saturated aqueous NaHCO_3_ solution (0.1 ml) and Et_2_O (1.5 ml). The reaction mixture was stirred at room temperature for 12 h under nitrogen atmosphere. After completion of the reaction, AcOEt (20 ml) was added, and the organic layer was washed with a saturated aqueous NaHCO_3_ solution (3 × 20 ml), dried over Na_2_SO_4_, filtered and concentrated under reduced pressure. TBAF (1.0 M in THF, 10 eq) was added dropwise to a solution of this crude in THF (3 ml) at 0 °C, and the reaction mixture was stirred at room temperature for 12 h. The reaction mixture was passed through Dowex^®^ 50WX8 (H^+^) column and eluted with H_2_O. The collected fractions were combined, concentrated and subjected to HPLC purification using a 4.6 × 150 mm Synergy Hydro C18 4 µm column and a mixture of MeOH/H_2_O (97:3), trifluoroacetic acid 0.1% as eluent at a flow rate of 0.5 ml/min. The process was monitored (diode array detector) at 280 nm. The chromatographic peaks were collected and concentrated, obtaining the titled compounds **4a**–**4c** as clear yellow oils.

#### (3*R*,3'*R*,4*S*,4'*S*,5*R*,5'*R*)-*N*,*N*'-(ethane-1,2-diyl)bis(3,4,5-trihydroxycyclohex-1-ene-1-carboxamide) (4a)

34 mg, 38% yield. IR (neat): ṽ = 3286, 2916, 1662, 1608, 1541, 1061 cm^−1^. [α]_D_ = −115.3 (*c* 1.0, H_2_O). ^1^H NMR (400 MHz, D_2_O): *δ* = 2.21 (dd, *J* = 17.7, 7.1 Hz, 2H, H-6), 2.73 (dd, *J* = 17.7, 5.3 Hz, 2H, H-6´), 3.44 (s, 4H, CH_2_N), 3.74 (dd, *J* = 8.7, 4.3 Hz, 2H, H-4), 3.99–4.06 (m, 2H, H-5), 4.42 (dd, *J* = 4.2, 4.2 Hz, 2H, H-3), 6.36–6.40 (m, 2H, H-2) ppm. ^13^C NMR (100 MHz, D_2_O): *δ* = 31.0 (C6), 39.0 (CH_2_N), 66.0 (C-4), 66.2 (C-5), 71.2 (C-3), 131.0 (C-2), 133.0 (C-1), 170.3 (C=O) ppm. HRMS (FAB^+^): calcd. for C_16_H_25_N_2_O_8_ [M + H]^+^, *m/z* 373.1611; found for [M + H]^+^, *m/z* 373.1579.

#### (3*R*,3'*R*,4*S*,4'*S*,5*R*,5'*R*)-*N*,*N*'-(propane-1,3-diyl)bis(3,4,5-trihydroxycyclohex-1-ene-1-carboxamide) (4b)

41 mg, 44% yield. IR (neat): ṽ = 3297, 2921, 1662, 1609, 1541, 1063 cm^−1^. [α]_D_ = −99.7 (*c* 1.0, H_2_O). ^1^H NMR (400 MHz, D_2_O): *δ* = 1.78 (quin, *J* = 6.7 Hz, 2H, CH_2_), 2.22 (dddd, *J* = 17.6, 7.1, 2.1, 1.4 Hz, 2H, H-6), 2.74 (dd, *J* = 17.6, 5.4 Hz, 2H, H-6´), 3.30 (t, *J* = 6.7 Hz, 4H, CH_2_N), 3.74 (dd, *J* = 8.7, 4.3 Hz, 2H, H-4), 3.99–4.05 (m, 2H, H-5), 4.42 (dd, *J* = 4.3, 4.3 Hz, 2H, H-3), 6.39–6.42 (m, 2H, H-2) ppm. ^13^C NMR (100 MHz, D_2_O): *δ* = 28.0 (CH_2_), 31.0 (C6), 37.0 (CH_2_N), 66.0 (C-4), 66.3 (C-5), 71.4 (C-3), 131.0 (C-2), 133.1 (C-1), 170.1 (C=O) ppm. HRMS (FAB^+^): calcd. for C_17_H_27_N_2_O_8_ [M + H]^+^, *m/z* 387.1767; found for [M + H]^+^, *m/z* 387.1771.

#### (3*R*,3'*R*,4*S*,4'*S*,5*R*,5'*R*)-*N*,*N*'-(butane-1,4-diyl)bis(3,4,5-trihydroxycyclohex-1-ene-1-carboxamide) (4c)

39 mg, 41% yield. IR (neat): ṽ = 3289, 2922, 1661, 1608, 1541, 1062 cm^−1^. [α]_D_ = −98.9 (*c* 1.0, H_2_O). ^1^H NMR (400 MHz, D_2_O): *δ* = 1.51–1.60 (m, 4H, CH_2_), 2.22 (dd, *J* = 17.6, 7.0 Hz, 2H, H-6), 2.74 (dd, *J* = 17.6, 5.2 Hz, 2H, H-6´), 3.24–3.30 (m, 4H, CH_2_N), 3.74 (dd, *J* = 8.7, 4.3 Hz, 2H, H-4), 3.99–4.07 (m, 2H, H-5), 4.42 (dd, *J* = 4.1, 4.1 Hz, 2H, H-3), 6.38–6.42 (m, 2H, H-2) ppm. ^13^C NMR (100 MHz, D_2_O): *δ* = 24.3 (CH_2_), 30.0 (C6), 38.0 (CH_2_N), 64.4 (C-4), 65.0 (C-5), 70.1 (C-3), 129.3 (C-2), 132.0 (C-1), 169.0 (C=O) ppm. HRMS (FAB^+^): calcd. for C_18_H_29_N_2_O_8_ [M + H]^+^, *m/z* 401.1924; found for [M + H]^+^, *m/z* 401.1902.

### Enzyme kinetics

#### Cloning of Escherichia coli aroE gene

Amplification of *aroE* gene from *E. coli* strain JM101^20^ was carried out using the pair of PCR primers 5′-GGGAATTCCATATGGAAACCTATGCTGTTTTTGG-3′ (forward) and 5′-GGTTGGGGATCCTCACGCGGACAATTCCTCCTG-3′ (reverse) in which the restriction sites for *Nde* I and *BamH* I respectively, are underlined. The PCR product was digested (Thermo Scientific, Waltman, MA) and ligated using the Zero Blunt TOPO PCR Cloning kit (Invitrogen, Carlsbad, CA). For protein overproduction, the gene was subcloned into plasmid pCold-I (Takara Bio Inc., Otsu, Shiga, Japan) containing a 6-Histidine tag at its N-terminus that can be removed by factor Xa protease; the resulting plasmid was called pCold-EcSDH. *E. coli* Δ*aroE*:: Kan^R^ (JW3242) from the Keio collection^21^ was transformed with pCold-EcSDH, and the clone was sequenced using local facilities (Unidad de Síntesis y Secuenciación-IBt, UNAM).

#### Enzyme expression and purification

Transformed bacteria were used to inoculate 50 ml of Luria–Bertani (LB) media supplemented with ampicillin (100 µg/ml) and kanamycin (30 µg/ml). Cultures were incubated at 37 °C at 300 rpm until it reached an optical density (OD_600nm_) of 3.5, then 1 l of LB was inoculated in a Fernbach flask. Cells were grown at 37 °C and shaken at 200 rpm until the OD_600nm_ reached 0.4 afterwards the culture was maintained at 15 °C for 30 min, and expression was induced with 0.5 mM of isopropyl thiogalactoside. The culture was allowed to grow with shaking for additional 17 h at 14 °C.

Cells were harvested by centrifugation at 8000 rpm for 10 min, washed with 20 ml of TBS (Tris-HCl 50 mM, NaCl 150 mM, pH 7.5) and resuspended in 50 ml of lysis buffer A (Tris-HCl 50 mM, NaCl 500 mM, pH 8.0) mixed with 1 mg/ml of lysozyme (Sigma-Aldrich, St Louis, MO). After 30 min of incubation on ice, the mixture was added with protease inhibitor PMSF (Sigma-Aldrich) to a final concentration of 1 mM. Cells were lysed by sonication using Sonics Vibra Cell (Cole-Parmer Instruments, Vernon Hills, IL) at an amplitude of 40% for 5 min. The lysate was centrifugated at 13,000 rpm for 30 min at 4 °C, and the supernatant containing His6-tagged EcSDH was loaded onto a 1 ml Ni^2+^ chelating HisTrap FF column (GE Healthcare Life Sciences, Pittsburgh, PA) previously equilibrated with 10 volumes of lysis buffer containing 20 mM imidazole. The protein was eluted with an increasing gradient of imidazole (20–250 mM) in lysis buffer at a flow rate of 2 ml/min and analysed by SDS-PAGE with Coomassie staining.

Fractions containing His6-tagged EcSDH were concentrated and dialysed against 10 volumes of cleavage buffer B (Tris-HCl 20 mM, NaCl 100 mM, CaCl_2_ 2 mM, pH 8.0) in an Amicon centrifugal filter device with a 10 kDa cut-off (Millipore Corporation, Billerica, MA). Subsequent tag cleavage with factor Xa protease (NEB Biolabs, Beverley, MA) was performed at a concentration of 0.002 mg/ml of fraction volume, and the reaction was shaken at 16 °C for 48 h. A second affinity column in buffer C (Tris-HCl 50 mM, NaCl 50 mM, pH 7.5) was employed to recover EcSDH and cleavage of the tag from the enzyme was verified by SDS-PAGE. Pooled EcSDH was concentrated as previously described and further purified by loading onto a 120 ml HiLoad 16/600 Superdex 75 pg column (GE Healthcare, Little Chalfont, Buckinghamshire, UK) coupled to an Äkta-Pure system (GE Healthcare) eluting with buffer C at a flow rate of 1 ml/min. Fractions corresponding to the largest peak were dialysed against buffer D (Tris-HCl 50 mM, NaCl 50 mM, DTT 0.4 mM, EDTA 0.1 mM, glycerol 50%, pH 7.5) before storage at −70 °C. Purification, dialysis and concentration procedures were performed at 4 °C. The Bradford method^22^ was used to determine the protein concentration using bovine serum albumin as standard.

#### Enzymatic activity assay

The enzymatic activity of EcSDH was assayed at 25 °C by monitoring the oxidation of NADPH at 340 nm (ϵ = 6.18 × 10^3^ M^−1 ^cm^−1^). All assays were performed in a Genesys 10 S UV-vis spectrophotometer (Thermo Fisher Scientific Inc., Waltham, MA). The reaction mix contained (final volume 200 µl) KH_2_PO_4_ 100 mM pH 7.5, NADPH 0.21 mM, DHS, synthesised molecules and diluted enzyme. Stored enzyme stocks (200 U/ml) were diluted 1:100 in KH_2_PO_4_ 100 mM pH 7.5 before use. The reaction was initiated by addition of 2 µl of the diluted EcSDH enzyme and absorbance was measured within 2 min. During assays NADPH concentration was fixed at 0.21 mM, DHS concentrations were 0.0625, 0.125, 0.25, 0.5, 1 and 1.5 mM and concentrations of amides were 0, 0.25, 0.5, 1 and 1.5 mM. Experiments were performed in triplicate.

To determine the inhibition mode Lineweaver–Burk plots of the inverse of velocities (1/*v*) versus the inverse of substrate concentration 1/[S] were obtained. The Michaelis–Menten constant (*K_m_*), maximum velocity (*V*_max_) and inhibition constants (*K_i_* and *K_I_*) were analysed using Equation (1)^22^^,^^23^. Where *I* represent the inhibitor concentration, *K_I_* is defined as the dissociation constant for the enzyme-substrate-inhibitor complex and *K_i_* as the dissociation constant for the enzyme-inhibitor complex. Kinetic data were processed by nonlinear regression using the Origin software (OriginLab, Northampton, MA).

Dissociation constants were related to *IC*_50_ values following the Cheng–Prusoff Equation (2)^24^. Where *IC*_50_ represents the concentration of inhibitor producing 50% of enzyme inhibition. The determined *K_m_* of DHS was 92.29 ± 2.70 µM, and 125 µM of DHS was delimited as the substrate concentration [S]:

(1) $$v=\frac{V_{max}\;[S]}{\left[S\right]\left(1+\left[I\right]/K_{I}\right)+\;K_{m}(1+\;\left[I\right]/K_{i})}$$

(2) $${IC}_{50}=\frac{\;K_{m}+[S]}{\left(K_{m}/K_{i}\right)+\;(\left[S\right]/K_{I})}.$$

### Minimal inhibitory concentration (MIC) determination

The broth microdilution method^25^ was used to determine the MIC of synthesised molecules against *E. coli* strain JM101. Cells were grown to exponential phase in LB media (OD_600nm_ ∼0.5) and the cultures were diluted to a concentration of 2 × 10^5^ colony-forming units/ml. The 96-well plates containing 75 µl of LB media and synthesised compounds were inoculated with 75 µl of the bacterial suspension and shaken at 37 °C for 16 h. Compounds were tested in a range of doubling concentrations between 4 and 512 mg/l with a final dimethylsulphoxide concentration of 0.8% (v/v). LB media supplemented with kanamycin was used as a positive control, and the OD_600nm_ of the DMSO-treated control was taken as representative of 100% bacterial growth. The MIC was defined as the lowest concentration of compound that completely inhibited bacterial growth. Experiments were performed in duplicate.

### Molecular docking studies

The crystal structure of EcSDH enzyme (chain A) was taken from the Protein Data Bank (PDB code: 1NYT). After removing water molecules and heteroatoms, the protein was prepared at a pH 7.4 and hydrogen atoms were added using *Protein Preparation Wizard*^26^ from the Schrödinger Software, suite 2014-3^27^. Energy minimisation on the enzyme was performed with YASARA^28^. Synthesised compounds were built and geometry optimised (MMFF94 force field) via the Molecular Docking Server^29^. A first docking was performed using the minimised protein structure, and the cofactor NADPH + H^+^ and position of the ligand was checked against that reported in the literature^30^. In a second step, the protein with the docked cofactor was used as a target to dock the synthesised compounds. Docking of the ligands were carried out using the Molecular Docking Server with default settings. A box around the binding pocket of the enzyme was delimited as the simulation zone. Two hundred and fifty-five runs were employed in docking calculations. Interactions were analysed and molecular models were built in PyMol^31^.

## Results and discussion

### Chemistry

**Scheme 1.**
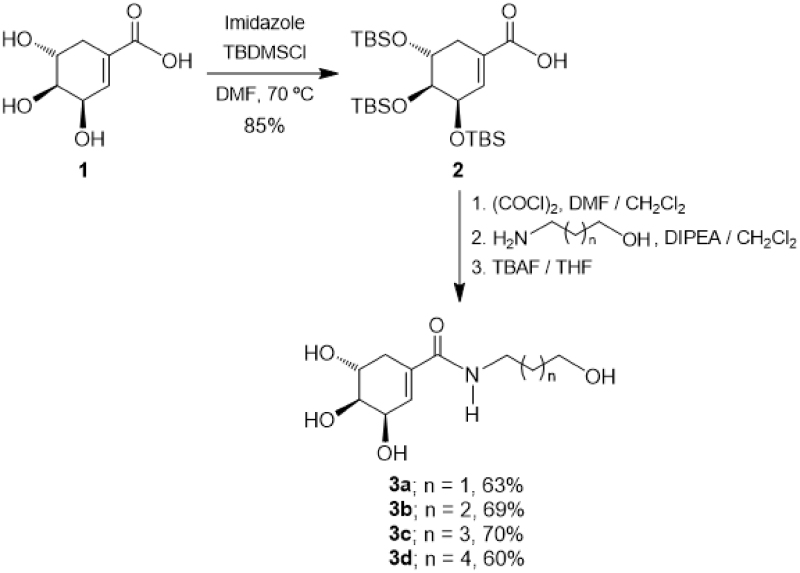
Synthesis of monoamides **3a–d**.

The synthesis of target monoamides **3a–3d** was achieved by a convenient procedure starting from the commercially available (-)-shikimic acid **1** as shown in Scheme 1. Thus, in the first step (-)-shikimic acid **1** was reacted with *tert*-butyldimethylsilyl chloride (TBDMSCl) in the presence of imidazole in DMF solution at 70 °C, obtaining the compound **2** with selective protection of the hydroxyl groups at C-3, C-4 and C-5 as *tert*-butyldimethylsilyl ethers in 85% yield^32^. Then, the acid function in the silylated derivative **2** was transformed into the corresponding acid chloride by treatment with oxalyl chloride in DMF/CH_2_Cl_2_, which by amidation with the appropriate hydroxylamine in the presence of DIPEA as a base in CH_2_Cl_2_^33^ followed by treatment with TBAF in THF, afforded the desired monoamides **3a–3d** in 60–70% yield^34^.

**Scheme 2.**
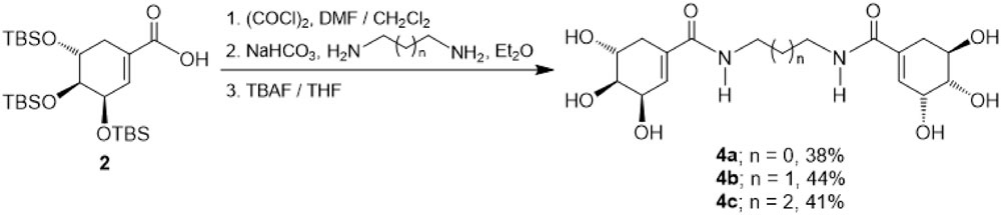
Synthesis of diamides **4a–c**.

On the other hand, diamides **4a–4c** which incorporates two units of SA were prepared through a slightly different procedure, as depicted in Scheme 2. Thus, the *O*-protected SA **2** was reacted with oxalyl chloride in DMF/CH_2_Cl_2_, and the intermediate acid chloride was treated with the corresponding diamine in Et_2_O in the presence of sodium bicarbonate (NaHCO_3_)^35^. Finally, the silyl group removal was performed by reaction with TBAF in THF, obtaining the diamides **4a–4c** in 38–44% yield.

### Biological activity

#### Enzyme inhibition assays

**Figure 1.**
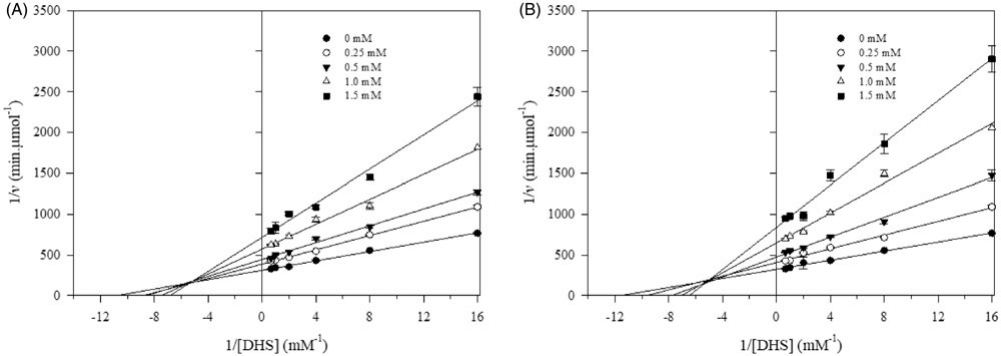
Lineweaver–Burk plots for inhibitory activity of mono and di amides against EcSDH. Lineweaver–Burk plots of EcSDH activity in the presence of (A) **3d** and (B) **4b**. Substrate DHS concentrations were 0.0625, 0.125, 0.25, 0.5, 1.0 and 1.5 mM, respectively. The data represent the average of three experiments.

The inhibitory effect of SA amide-derivatives was tested against EcSDH at pH 7.4. To characterise the enzymatic activity for its more biological meaningful reaction, the enzyme was assayed in the direction of SA formation, for this DHS was used as the substrate. The activity of EcSDH enzyme was determined in the presence of different concentrations of synthesised compounds, and Lineweaver–Burk plots of (1/v) versus 1/[S] were obtained. Representative inhibition plots of amides are shown in Figure 1. The double reciprocal plots of all tested compounds showed a family of the second quadrant intersecting lines indicative of mixed type inhibition concerning DHS^36^. The mechanism underlying mixed type inhibition implies that inhibitor may bind to the ligand-free and ligand bounded enzyme but exhibit different affinities for one state or the other. To characterise the kinetic parameters, data were analysed using Equation (1). The inhibition constants (*K_i_* and *K_I_*) are summarised in Table 1. For every synthesised amide *K_i_* < *K_I_* indicating a stronger affinity for the substrate free instead of bounded substrate enzyme. Monoamides displayed inhibition with *K_i_* values in the 587–733 µM range (Table 1). Despite the similar magnitude of inhibition, it appears that inhibition activity smoothly increases with increase in the alkyl chain length. The weakest inhibitor is corresponding to that of the propanol moiety in **3a** (*K_i_*= 733 ± 67 µM) compared to the hexanol derivative **3d** (*K_i_*= 587 ± 49 µM). Among the evaluated compounds, diamides were the most active inhibitors (*K_i_* = 400–458 µM) (Table 1). The similar inhibitory effect of diamides may arise with strong participation of the extra SA substituent. According to our results, the structural variations of synthesised compounds resulted in two inhibition potencies, *IC*_50_= 829–953 µM for monoamides and *IC*_50_= 588–652 µM for diamides. This behaviour may suggest two different binding modes to the enzyme.

**Table 1.** Kinetic data and MIC values for the inhibitory effects of synthesised compounds.

Compound No.	*K_i_* (µM)	*K_I_* (µM)	*IC*_50_ (µM)	MIC (mg/l)
**Mon3**	733 ± 67	1214 ± 58	949 ± 33	512
**Mon4**	717 ± 71	1145 ± 130	882 ± 54	512
**Mon5**	642 ± 34	1483 ± 91	953 ± 10	512
**Mon6**	587 ± 49	1188 ± 26	829 ± 49	512
**Dim2**	416 ± 69	831 ± 12	588 ± 55	512
**Dim3**	400 ± 50	896 ± 72	589 ± 56	512
**Dim4**	458 ± 81	945 ± 13	652 ± 65	512

Our amide SA derivatives showed weak inhibitory activity against EcSDH (*K_i_* = 400–737 µM) compared to the starting SA (*K_i_* = 160 µM) or the aldehyde vainillin (*K_i_* = 93 µM) but similar to the related aromatic compounds gallic acid (*K_i_* = 380 µM) and protocatechuic acid (*K_i_* = 750 µM) tested in *P. sativum*^13^. We propose that the bioisosteric replacement of the carboxylic acid of SA for the N-substituted amides lead to weaker inhibitors which could be the result of either differences in electronic distribution (anion carboxylate versus neutral amides), geometry and steric hindrance of the derivatives and/or different binding modes to the enzyme.

#### Antibacterial activity of synthesised compounds

The compounds **3a**–**4c** were evaluated for growth inhibition of *E. coli* JM101. Although two groups of *IC*_50_ values were obtained in kinetic experiments, all molecules displayed the same weak antibacterial activity (MIC =512 mg/l, Table 1). It has been reported that *E. coli* possess at least one SA transporter^37^, as well as the machinery for transport of diamides^38^^,^^39^ and N-alkylated amides^40^. Also other polyhydroxylated six ring seemingly structures have been identified as SDH inhibitors active against Gram-negative bacterial growth^41^^,^^42^. Considering this observation and although our SA-derivatives bear structural modifications, it seems not possible that MIC values are due to negligible permeation of the cell.

### Docking studies

**Figure 2.**
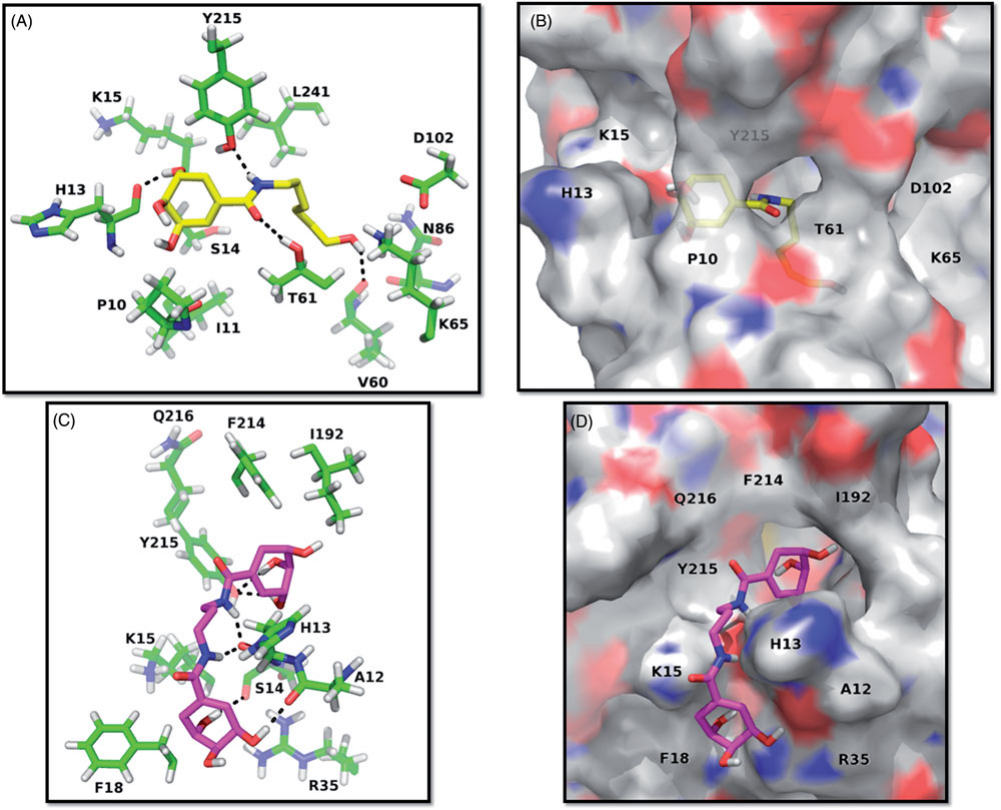
Predicted binding modes of mono and di amides to EcSDH. (A, C) Three-dimensional representations of EcSDH in complex with **3d** (yellow carbons) and **4b** (magenta carbons). The most involved residues are labelled and shown as green carbons. Hydrogen bonds are represented as black dashed lines. (B, D) Surface and stick representations of the proposed binding modes of synthesised amides.

Molecular docking studies were performed to rationalise the inhibitory activity of synthesised compounds by predicting their interactions with the active site of EcSDH enzyme. Taking into account that for all tested amides *K_i_* < *K_I_* the molecular binding modes to DHS-free enzyme were simulated. Analysis of monoamides predicted that they could all bind to the active site in a similar fashion. Interactions originate from the hydroxyl ended alkyl chain, amide group an SA moiety (Figure 2(A,B)). Interestingly, the alkyl chain orients towards a hydrophilic patch inside the active site. Except for **3a,** all monoamides form one hydrogen bond between the hydroxyl group of their aliphatic chains and an amino acid of the hydrophilic patch. The amide group is held in place by hydrogen bonding of the N-H and C=O groups to the side chain of the conserved Y215 and the backbone of T61, respectively. Also, a hydrogen bond is formed between the hydroxyl group (C3) of the SA moiety and the backbone of H13. In the case of compounds **4a**–**4c** modelling suggested that one SA moiety binds to the active site of EcSDH whereas the amide groups and the second SA moiety anchors to a hydrophilic groove in the immediacy of the binding pocket (Figure 2(C,D)), which is in good agreement with the observed inhibition modes. Inside the active site, the side chain of Y215 and hydroxyl groups of the SA moiety form hydrogen bonds. Furthermore, the amide groups create hydrogen bonds with H13. Finally, in the vicinity of the binding pocket hydroxyl groups of the SA nucleus interact with the backbones of S14 and A12 via hydrogen bonding. All described hydrogen bonds were in the 1.8–2.4 Å range of distance.

The docking studies suggest that major determinants for binding of synthesised amides are the hydroxyl groups of SA moieties or N-alkylated chains and the amide groups. A hydrophilic patch in the active site of EcSDH was identified, it comprises the catalytic dyad K65-D102^12^ responsible for enzyme activity, as well as the side chains of N86, D102, Y215 and the backbone of V60 and T61. A hydrophilic groove in the immediacy of the binding pocket is composed of the backbones of A12, H13, S14 and the side chain of R35. These observations highlight two potential points of interaction for binding of polar molecules as polyhydroxylated non-bulky derivatives that can be explored in the development of compounds with inhibitory activity.

Bioisosteric replacement of the carboxylate moiety for SA amide derivatives lead to weaker inhibitors than the starting acid. Molecular modelling studies suggested that although sharing interactions with amino acids S14, T61 and Y215 the synthesised compounds bind to somewhat more superficial subsites in the EcSDH enzyme compared to SA. The prediction suggested that SA binds deeply inside the catalytic pocket and protein crystal structures of SDH enzymes with SA (PDB code= 3PHH, 3PHI and 3DOO) showed a similar buried binding. This subsite may be strongly related to catalytic activity as the competitive inhibitor vanillin is predicted to position there as well (Figure 3S).

## Conclusions

We have described a facile method for the preparation of amide derivatives of SA from simple available starting materials. In the present study, synthesised compounds **3a–4c** were evaluated for their *in vitro* EcSDH inhibition and antibacterial activity against *E. coli* JM101. Diamide derivatives proved to be stronger inhibitors of EcSDH enzyme than monoamides. The most active compounds were molecules **4a** and **4b** with *IC*_50_ 588 and 589 µM, respectively. Enzyme kinetic analysis showed that all tested compounds are mixed type inhibitors. Molecular modelling studies of compounds **3a–4c** suggested two different binding modes of monoamide and diamide compounds to the EcSDH active site. To the best of our knowledge, no previous studies have been reported on the possible inhibitory potential of SA derivatives as SDH inhibitors. These inhibitors represent useful chemical scaffolds that can be further modified for the development of novel SDH inhibitors with enhanced inhibitory activity.

## Supplementary Material

IENZ_1422125_Supplementary_Material.pdf

## References

[1] Mir R, Jallu S, Singh TP. The shikimate pathway: review of amino acid sequence, function and three-dimensional structures of the enzymes. Crit Rev Microbiol 2015;41:172–89. 23919299 10.3109/1040841X.2013.813901

[2] Maeda H, Dudareva N. The shikimate pathway and aromatic amino acid biosynthesis in plants. Annu Rev Plant Biol 2012;63:73–105. 22554242 10.1146/annurev-arplant-042811-105439

[3] Diaz Quiroz DC, Carmona SB, Bolivar F, Escalante A. Current perspectives on applications of shikimic and aminoshikimic acids in pharmaceutical chemistry. Res Rep Med Chem 2014;4:35–46.

[4] Martínez JA, Bolívar F, Escalante A. Shikimic acid production in *Escherichia coli*: from classical metabolic engineering strategies to omics applied to improve its production. Front Bioeng Biotechnol 2015;3:145. 26442259 10.3389/fbioe.2015.00145 PMC4585142

[5] Amer FA, El-behedy EM, Mohtady HA. New targets for antibacterial agents. Biotechnol Mol Biol Rev 2008;3:46–57.

[6] Gordon S, Simithy J, Goodwin D. Selective *Mycobacterium tuberculosis* shikimate kinase inhibitors as potential antibacterials. Perspect Medicin Chem 2015;7:9–20. 25861218 10.4137/PMC.S13212 PMC4362912

[7] Gonzalez-Bello C. Inhibition of shikimate kinase and type II dehydroquinase for antibiotic discovery: structure-based design and simulation studies. Curr Top Med Chem 2016;16:960–77. 26303426 10.2174/1568026615666150825142527

[8] Derrer B, Macheroux P, Kappes B. The shikimate pathway in apicomplexan parasites: implications for drug development. Front Biosci 2013;18:944. 10.2741/4155 23747859

[9] Roberts F, Roberts CW, Johnson JJ, et al Evidence for the shikimate pathway in apicomplexan parasites. Nature 1998;393:801. 9655396 10.1038/31723

[10] Steinrücken HC, Amrhein N. The herbicide glyphosate is a potent inhibitor of 5-enolpyruvyl-shikimic acid-3-phosphate synthase. Biochem Biophys Res Commun 1980;94:1207–12. 7396959 10.1016/0006-291x(80)90547-1

[11] Bai SH, Ogbourne SM. Glyphosate: environmental contamination, toxicity and potential risks to human health via food contamination. Environ Sci Pollut Res Int 2016;23:18988–9001. 27541149 10.1007/s11356-016-7425-3

[12] Peek J, Christendat D. The shikimate dehydrogenase family: functional diversity within a conserved structural and mechanistic framework. Arch Biochem Biophys 2015;566:85–99. 25524738 10.1016/j.abb.2014.12.006

[13] Balinsky D, Davies DD. Aromatic biosynthesis in higher plants. 2. Mode of attachment of shikimic acid and dehydroshikimic acid to dehydroshikimic reductase. Biochem J 1961;80:296–300. 13686343 10.1042/bj0800296 PMC1243997

[14] Baillie AC, Corbett JR, Dowsett JR, McCloskey P. Inhibitors of shikimate dehydrogenase as potential herbicides. Pestic Sci 1972;3:113–20. 10.1042/bj1260021pa PMC1178471 5075246

[15] Han C, Wang L, Yu K, et al Biochemical characterization and inhibitor discovery of shikimate dehydrogenase from *Helicobacter pylori*. FEBS J 2006;273:4682–92. 16972983 10.1111/j.1742-4658.2006.05469.x

[16] Peek J, Shi T, Christendat D. Identification of novel polyphenolic inhibitors of shikimate dehydrogenase (AroE). J Biomol Screen 2014;19:1090–8. 24632659 10.1177/1087057114527127

[17] Avitia-Domínguez C, Sierra-Campos E, Salas-Pacheco J, et al Inhibition and biochemical characterization of methicillin-resistant *Staphylococcus aureus* shikimate dehydrogenase: an *in silico* and kinetic study. Molecules 2014;19:4491–509. 24727420 10.3390/molecules19044491 PMC6270726

[18] Hsu K-C, Cheng W-C, Chen Y-F, et al Pathway-based screening strategy for multitarget inhibitors of diverse proteins in metabolic pathways. PLoS Comput Biol 2013;9:e1003127. 23861662 10.1371/journal.pcbi.1003127 PMC3701698

[19] Dell KA, Frost JW. Identification and removal of impediments to biocatalytic synthesis of aromatics from D-glucose: rate-limiting enzymes in the common pathway of aromatic amino acid biosynthesis. J Am Chem Soc 1993;115:11581–9.

[20] Flores N, Flores S, Escalante A, et al Adaptation for fast growth on glucose by differential expression of central carbon metabolism and gal regulon genes in an *Escherichia coli* strain lacking the phosphoenolpyruvate: carbohydrate phosphotransferase system. Metab Eng 2005;7:70–87. 15781417 10.1016/j.ymben.2004.10.002

[21] Baba T, Ara T, Hasegawa M, et al Construction of *Escherichia coli* K-12 in-frame, single-gene knockout mutants: the Keio collection. Mol Syst Biol 2006;2:2006.0008. 10.1038/msb4100050 PMC1681482 16738554

[22] Bradford MM. A rapid and sensitive method for the quantitation microgram quantities of protein utilizing the principle of protein-dye binding. Anal Biochem 1976;72:248–54. 942051 10.1016/0003-2697(76)90527-3

[23] Alimoradi N, Ashrafi-kooshk MR, Shahlaei M, et al Diethylalkylsulfonamido (4- methoxyphenyl)methyl) phosphonate/phosphonic acid derivatives act as acid phosphatase inhibitors: synthesis accompanied by experimental and molecular modeling assessments. J Enzyme Inhib Med Chem 2017;32:20–8. 27766897 10.1080/14756366.2016.1230109 PMC6010023

[24] Cheng Y-C, Prusoff WH. Relationship between the inhibition constant (K1) and the concentration of inhibitor which causes 50 per cent inhibition (I50) of an enzymatic reaction. Biochem Pharmacol 1973;22:3099–108. 4202581 10.1016/0006-2952(73)90196-2

[25] Andrews JM. Determination of minimum inhibitory concentrations. J Antimicrob Chemother 2001;48(suppl 1):5. 11420333 10.1093/jac/48.suppl_1.5

[26] Sastry GM, Adzhigirey M, Sherman W. Protein and ligand preparation: parameters, protocols, and influence on virtual screening enrichments. J Comput Aided Mol Des 2013;27:221–34. 23579614 10.1007/s10822-013-9644-8

[27] Schrödinger Maestro. LLC, New York, NY; 2014.

[28] Krieger E, Koraimann G, Vriend G. Increasing the precision of comparative models with YASARA NOVA – a self-parameterizing force field. Proteins 2002;47:393–402. 11948792 10.1002/prot.10104

[29] Bikadi Z, Hazai E. Application of the PM6 semi-empirical method to modeling proteins enhances docking accuracy of AutoDock. J Cheminform 2009;1:1–16. 20150996 10.1186/1758-2946-1-15 PMC2820493

[30] Michel G, Roszak AW, Sauvé V, et al Structures of shikimate dehydrogenase AroE and its paralog YdiB: a common structural framework for different activities. J Biol Chem 2003;278:19463–72. 12637497 10.1074/jbc.M300794200

[31] Schrödinger L. The PyMOL Molecular Graphics System, Version 1.8. 2015.

[32] Sánchez-Abella L, Fernández S, Verstuyf A, et al Synthesis, conformational analysis, and biological evaluation of 19- nor -vitamin D3 analogues with A-ring modifications. J Med Chem 2009;52:6158–62. 19739672 10.1021/jm900711d

[33] Ramkumar N, Raghavendra MS, Nagarajan R. Friedel–Crafts cyclodehydration approach toward the synthesis of ellipticine and 9-methoxyellipticine. SYNLETT 2014;25:2791–3.

[34] Sánchez-Abella L, Fernández S, Verstuyf A, et al Synthesis and biological evaluation of new 6-s-cis locked 1,2,25-trihydroxyprevitamin D3 analogues. Bioorg Med Chem 2007;15:4193–202. 17412598 10.1016/j.bmc.2007.03.058

[35] Sumiyoshi T, Nishimura K, Nakano M, et al Molecular assembly of C2-symmetric r, ω -alkylidenediamines into coiled coil and twisted ribbon aggregates. JACS 2003;125:12137–42. 10.1021/ja035085t 14519000

[36] Segel IH. Enzyme kinetics: behaviour and analysis of rapid equilibria and steady-state equilibria. New York: John Wiley and Sons; 1993.

[37] Whipp MJ, Camakaris H, Pittard AJ. Cloning and analysis of the shiA gene, which encodes the shikimate transport system of *Escherichia coli* K-12. Gene 1998;209:185–92. 9524262 10.1016/s0378-1119(98)00043-2

[38] Prabhala BK, Aduri NG, Jensen JM, et al New insights into the substrate specificities of proton-coupled oligopeptide transporters from *E. coli* by a pH sensitive assay. FEBS Lett 2014;588:560–5. 24440353 10.1016/j.febslet.2014.01.004

[39] Harder D, Stolz J, Casagrande F, et al DtpB (YhiP) and DtpA (TppB, YdgR) are prototypical proton-dependent peptide transporters of *Escherichia coli*. FEBS J 2008;275:3290–8. 18485005 10.1111/j.1742-4658.2008.06477.x

[40] Payne JW. Peptide transport in *Escherichia coli*: permease specificity towards terminal amino group substituents. J Gen Microbiol 1974;80:269–76. 4595007 10.1099/00221287-80-1-269

