# Supplementary material for: Synthesis, biological activity and molecular modelling studies of shikimic acid derivatives as inhibitors of the shikimate dehydrogenase enzyme of Escherichia coli
Source: J Enzyme Inhib Med Chem. 2018 Jan 24;33(1):397–404. doi: 10.1080/14756366.2017.1422125 (PMC6009893; doi:10.1080/14756366.2017.1422125)

SUPPLEMENTARY DATA FOR

Synthesis, biological activity and molecular modelling studies of shikimic acid derivatives as inhibitors of the shikimate dehydrogenase enzyme of *Escherichia coli*

Dulce Catalina Díaz-Quiroz, César Salvador Cardona-Félix, José Luis Viveros-Ceballos, Miguel Angel Reyes-González, Francisco Bolívar, Mario Ordoñez & Adelfo Escalante*

*Corresponding author

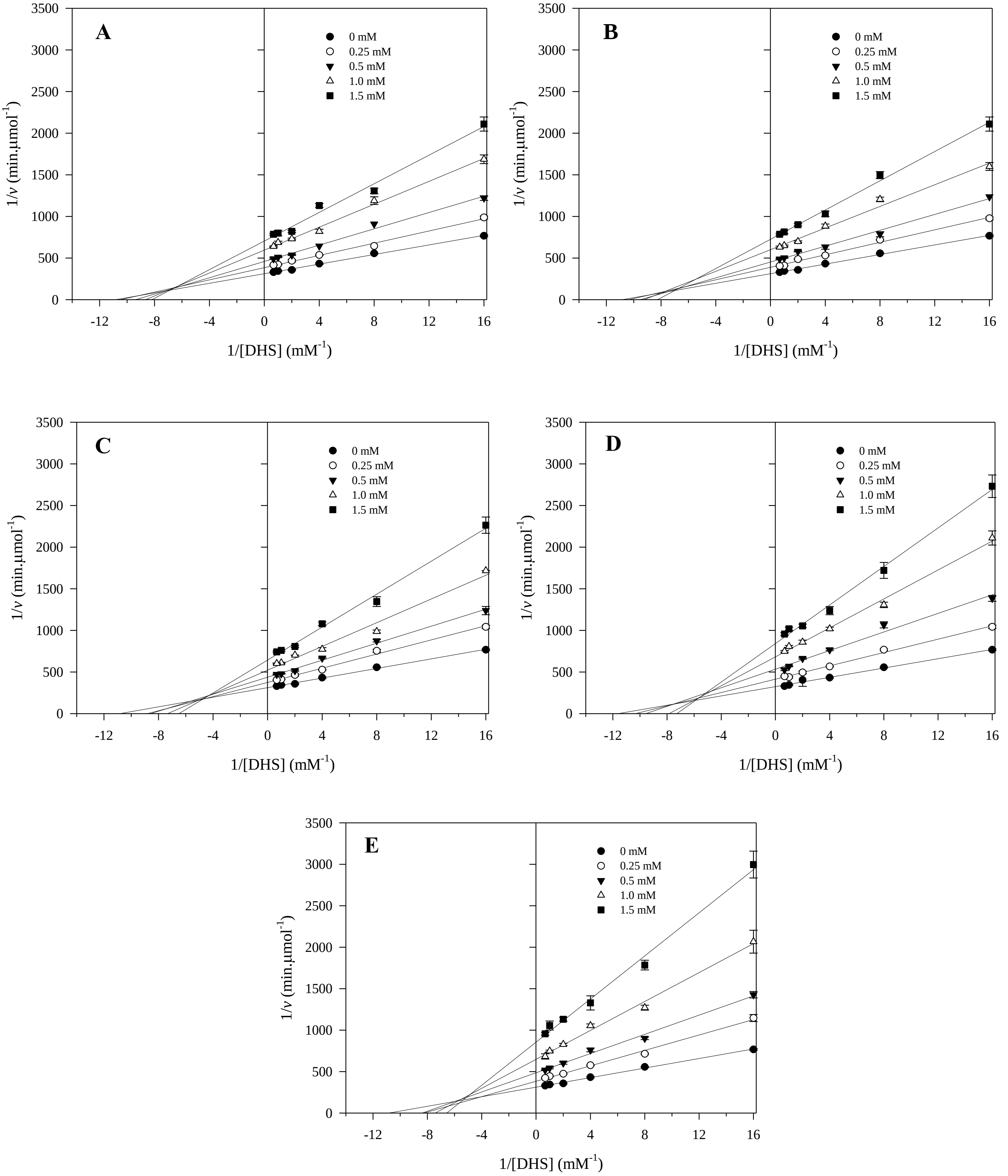

Figure 1S. Lineweaver-Burk plots of EcSDH activity in the presence of mono and diamides. **3a** (A), **3b** (B) and **3c** (C) or diamides **4a** (D) and **4c** (E). Substrate DHS concentrations were 0.0625, 0.125, 0.25, 0.5, 1.0 and 1.5 mM, respectively. The data represent the average of 3 experiments.

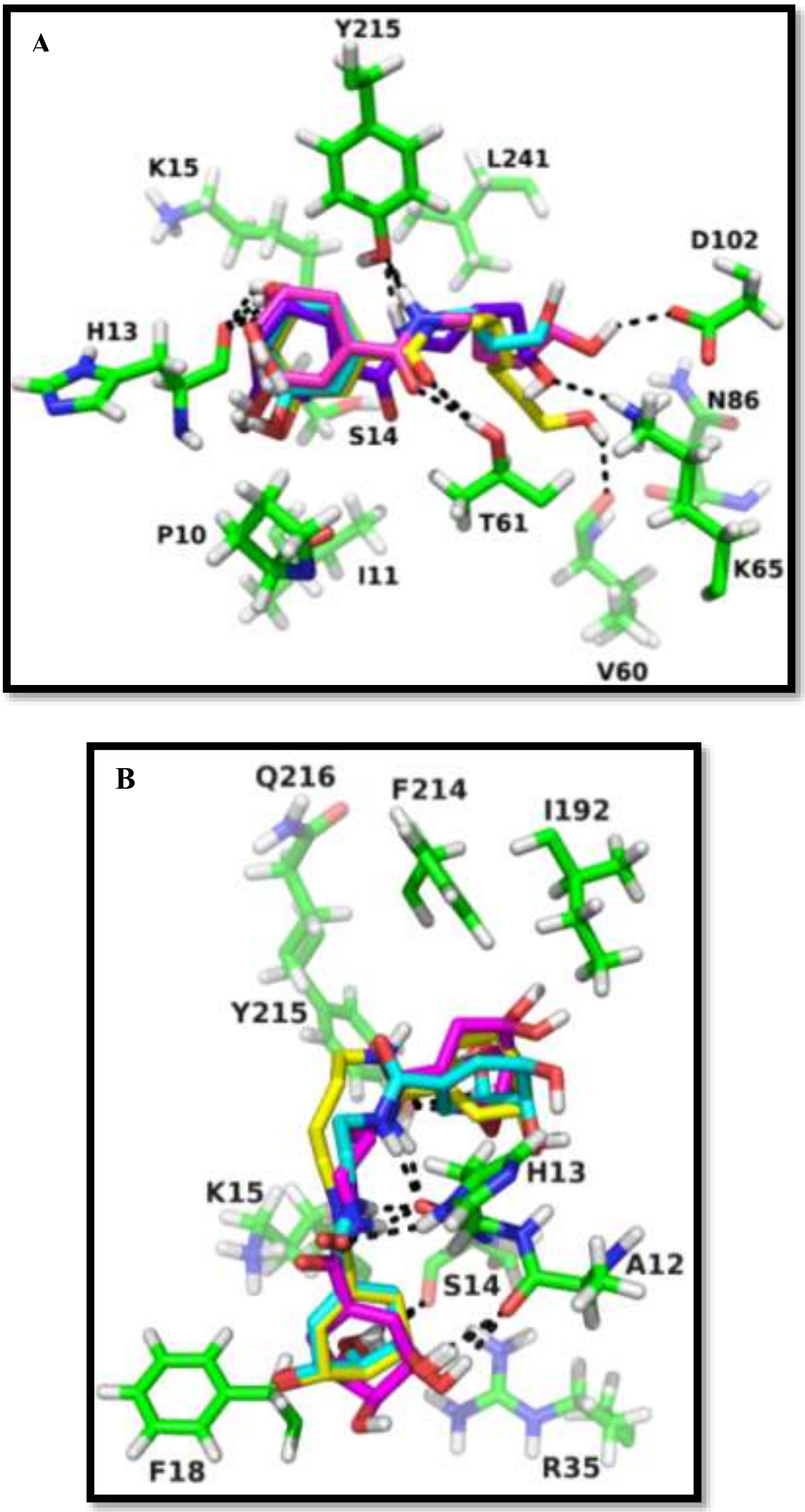

Figure 2S. Predicted binding modes of mono and di amides to EcSDH. Panel A: three-dimensional representations of EcSDH in complex with monoamides **3a** (cyan), **3b** (purple), **3c** (pink) and **3d** (yellow). B: Predicted binding modes of diamides **4a** (cyan), **4b** (magenta) and **4c** (yellow). The most involved residues are labeled and shown as green carbons. Hydrogen bonds are represented as black dashed lines.

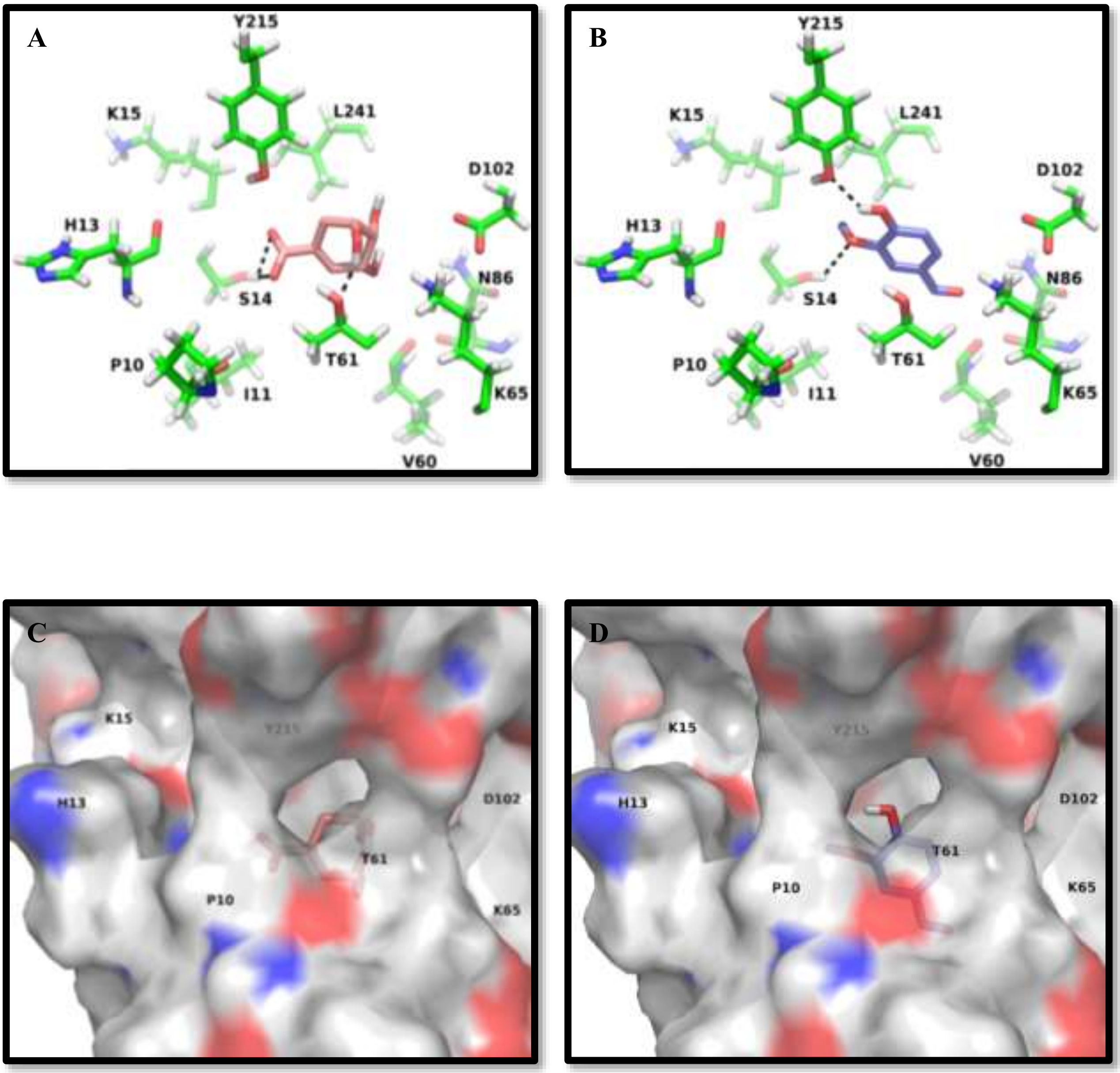

Figure 3S. Predicted binding modes of SA and vanillin to EcSDH. Panel A and B: three-dimensional representations of EcSDH in complex with SA (pink) and vanillin (slate) respectively. Panel C and D: Surface and stick representations of SA and vanillin binding modes to the enzyme. The most involved residues are labelled and shown as green carbons. Black dashed lines represent hydrogen bonds.

Compound **2** : **^{1}H-NMR**

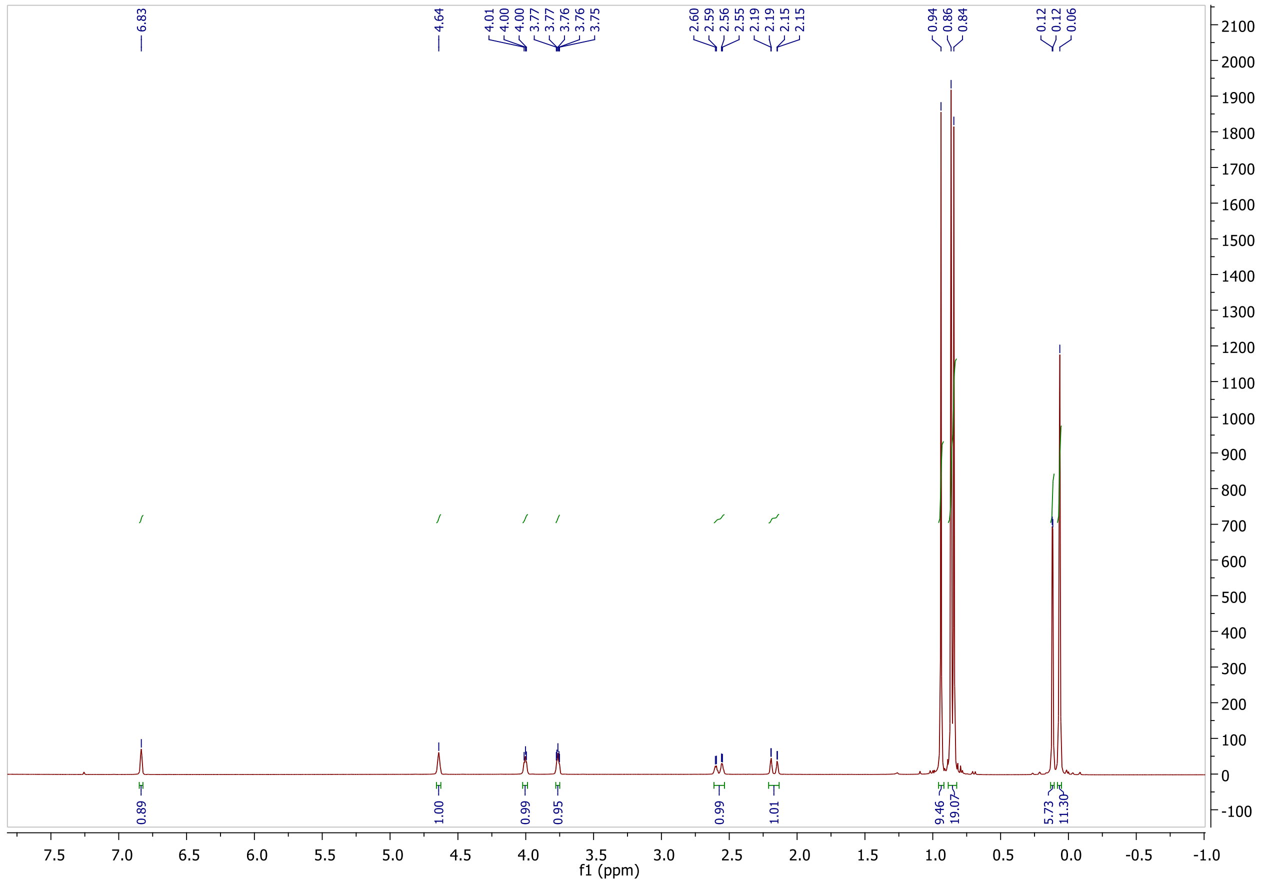

^{13}C-NMR

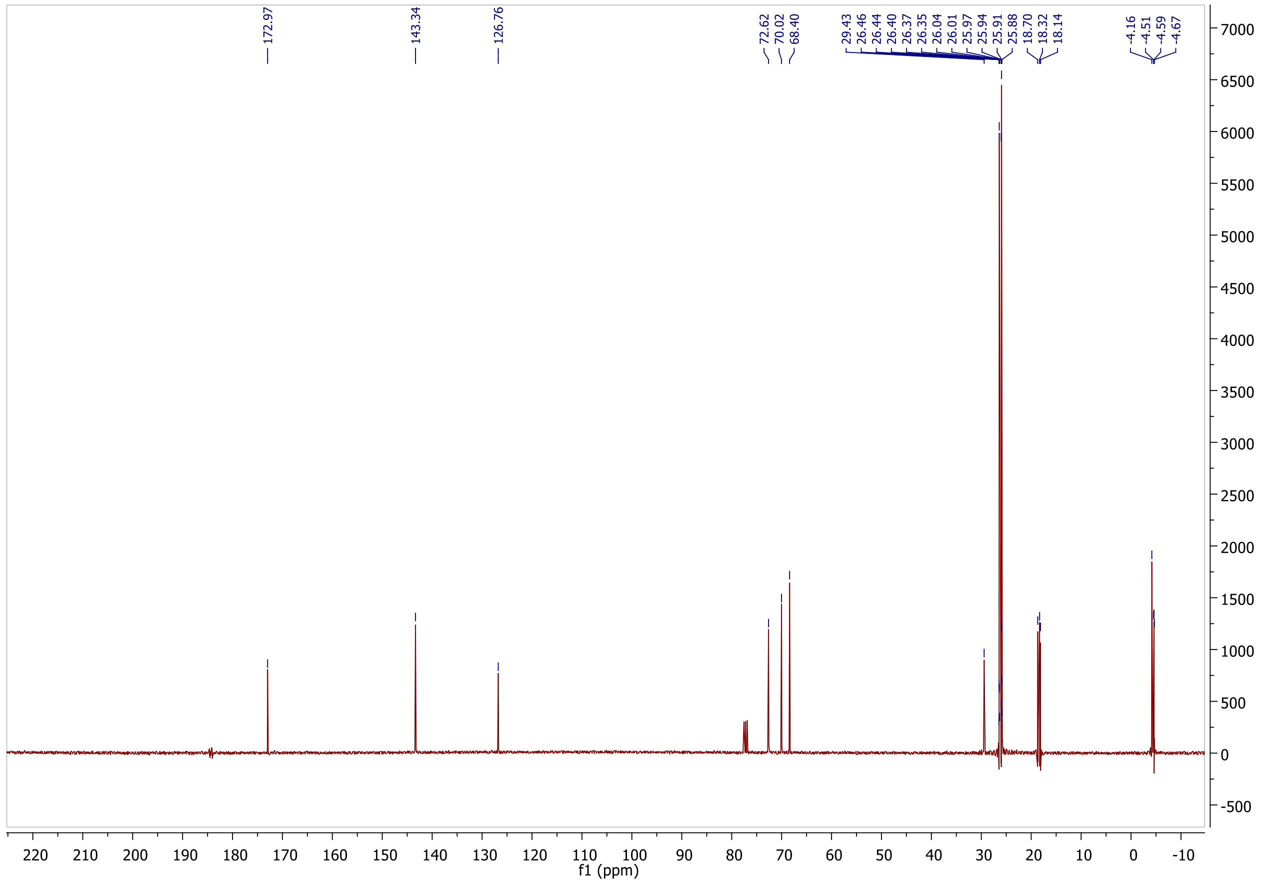

Compound **3a:** **^{1}H-NMR**

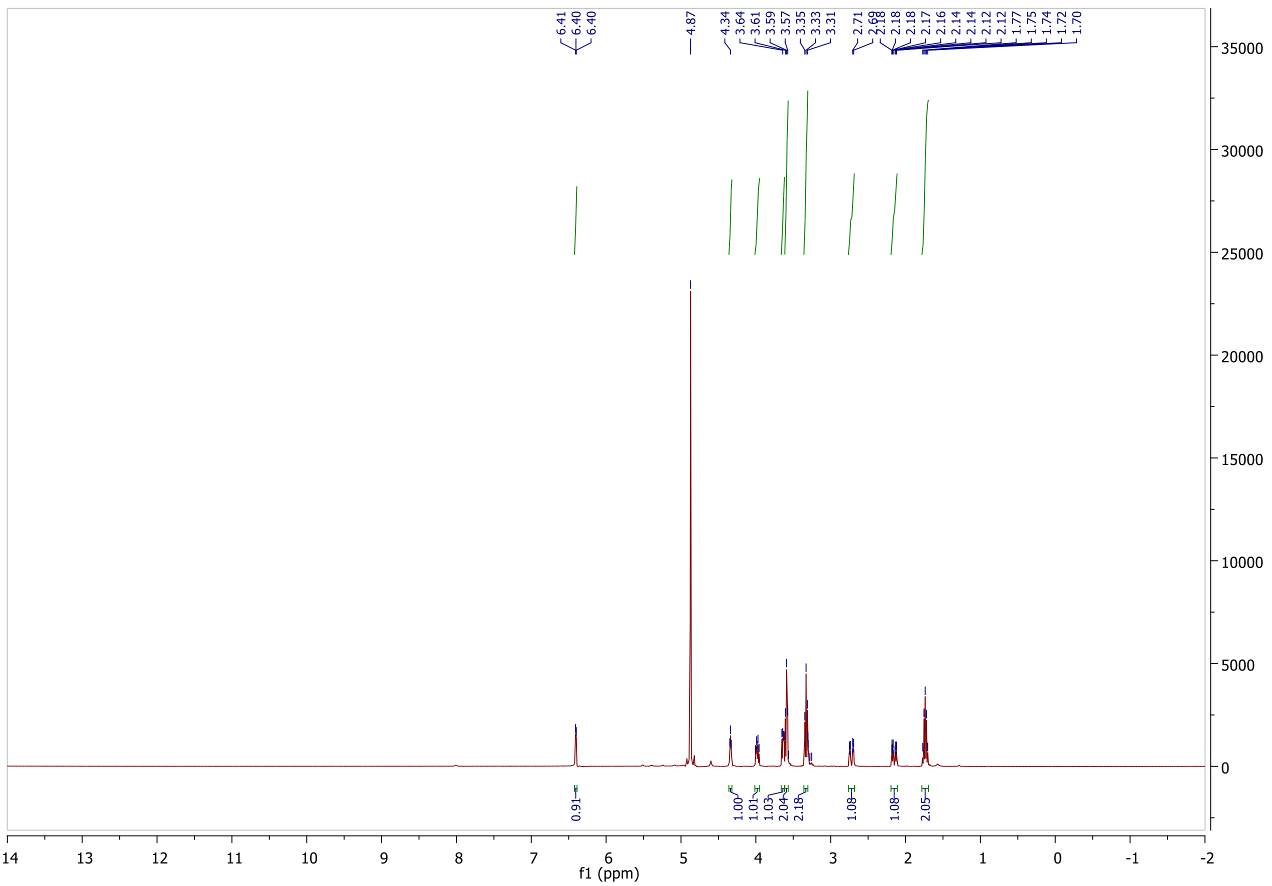

^{13}C-NMR

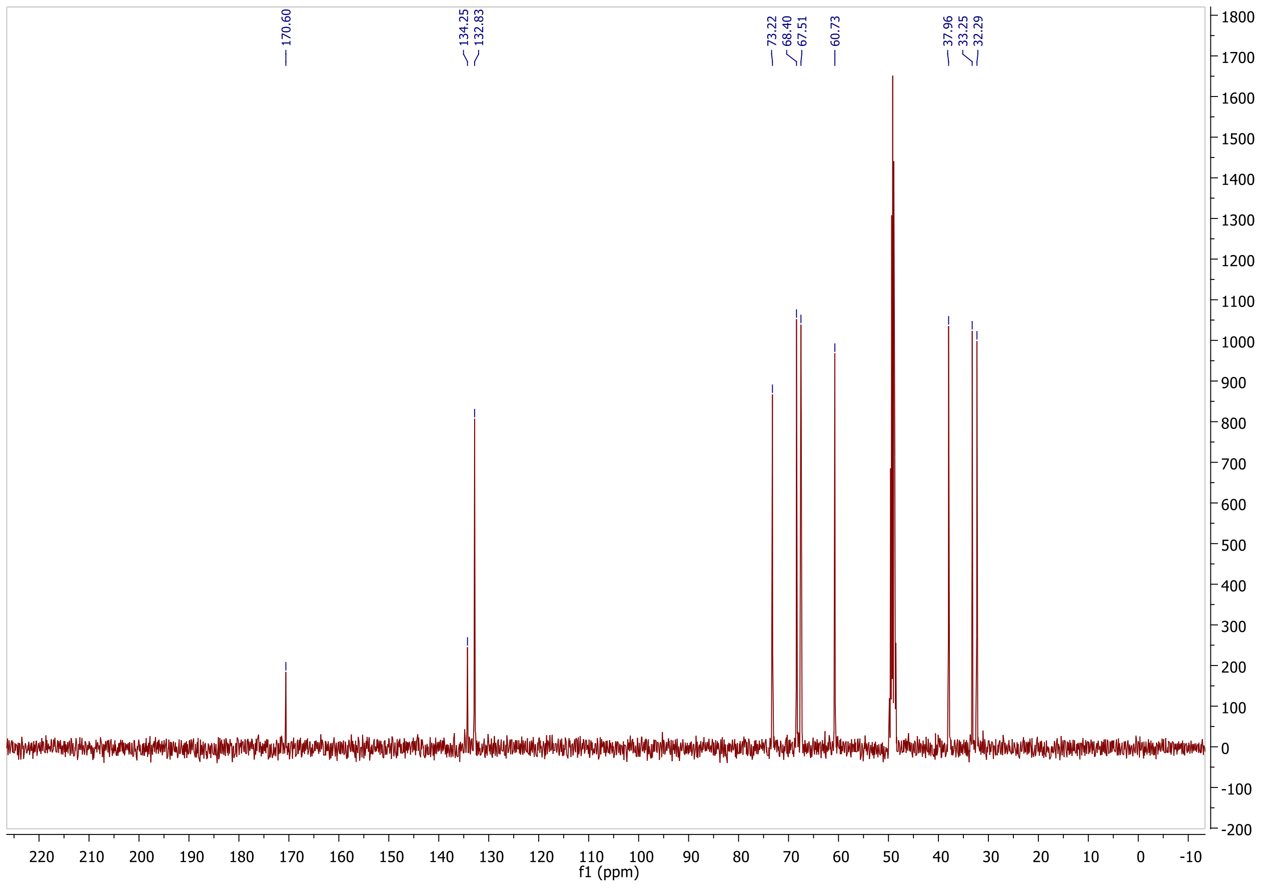

Compound **3b:** ^{1}**H-NMR**

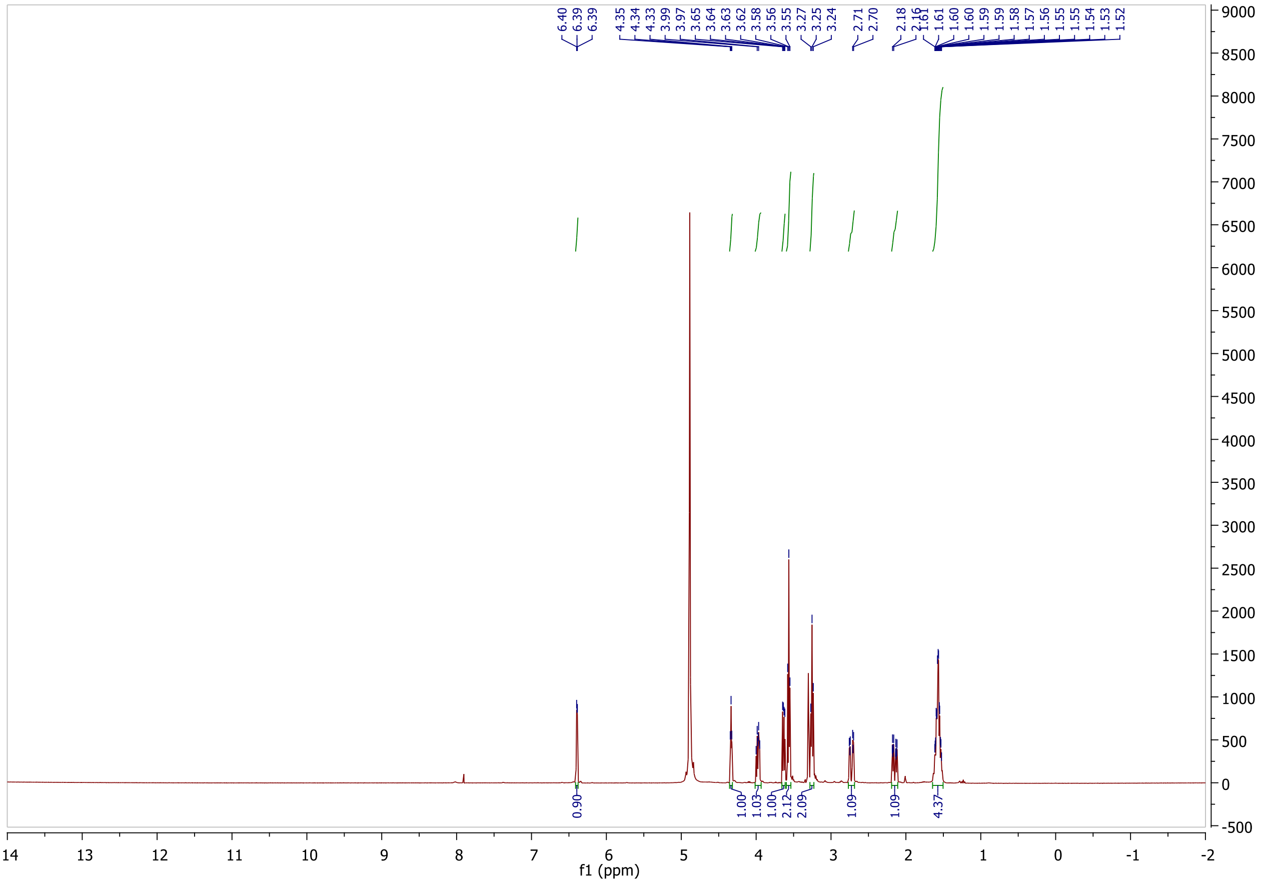

^{13}**C-NMR**

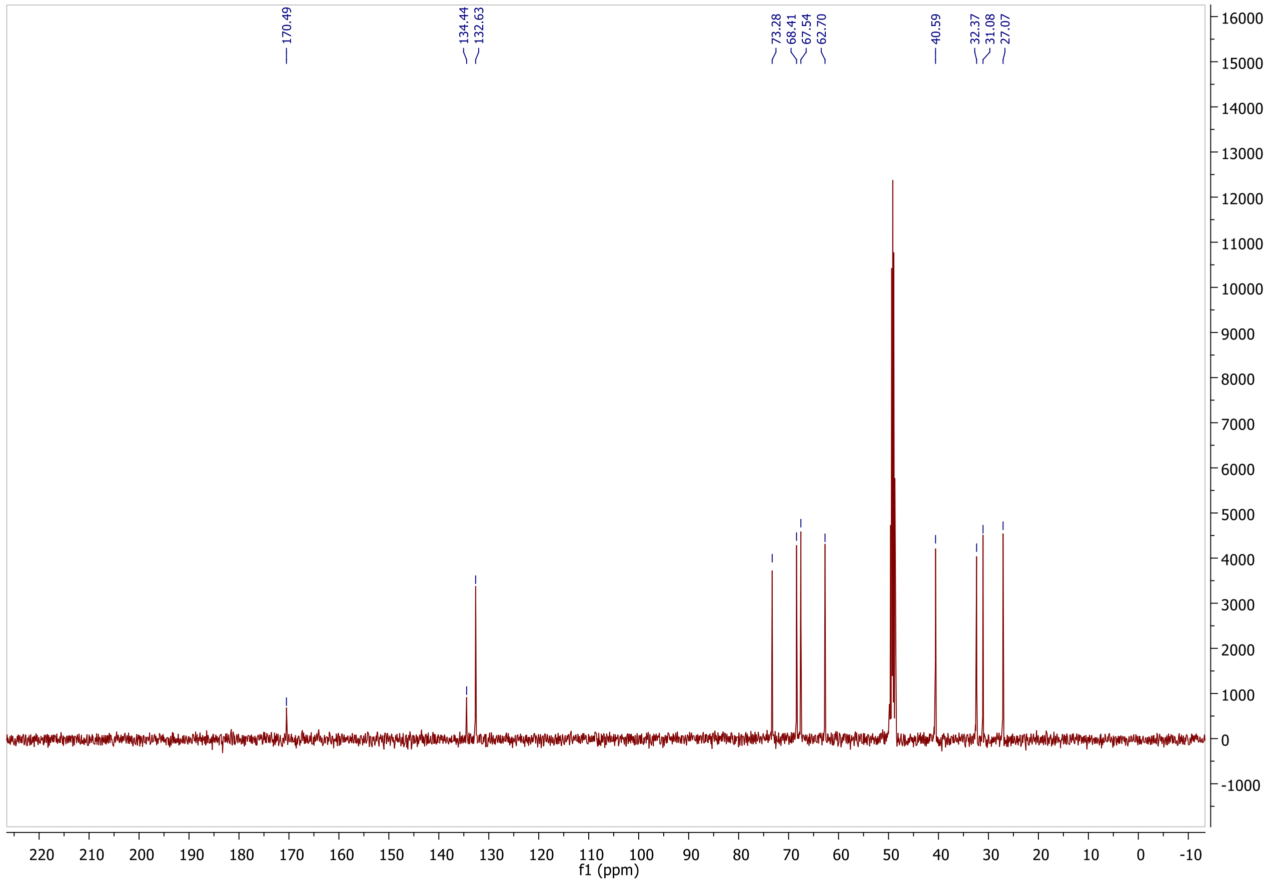

Compound **3c:** ^{1}**H-NMR**

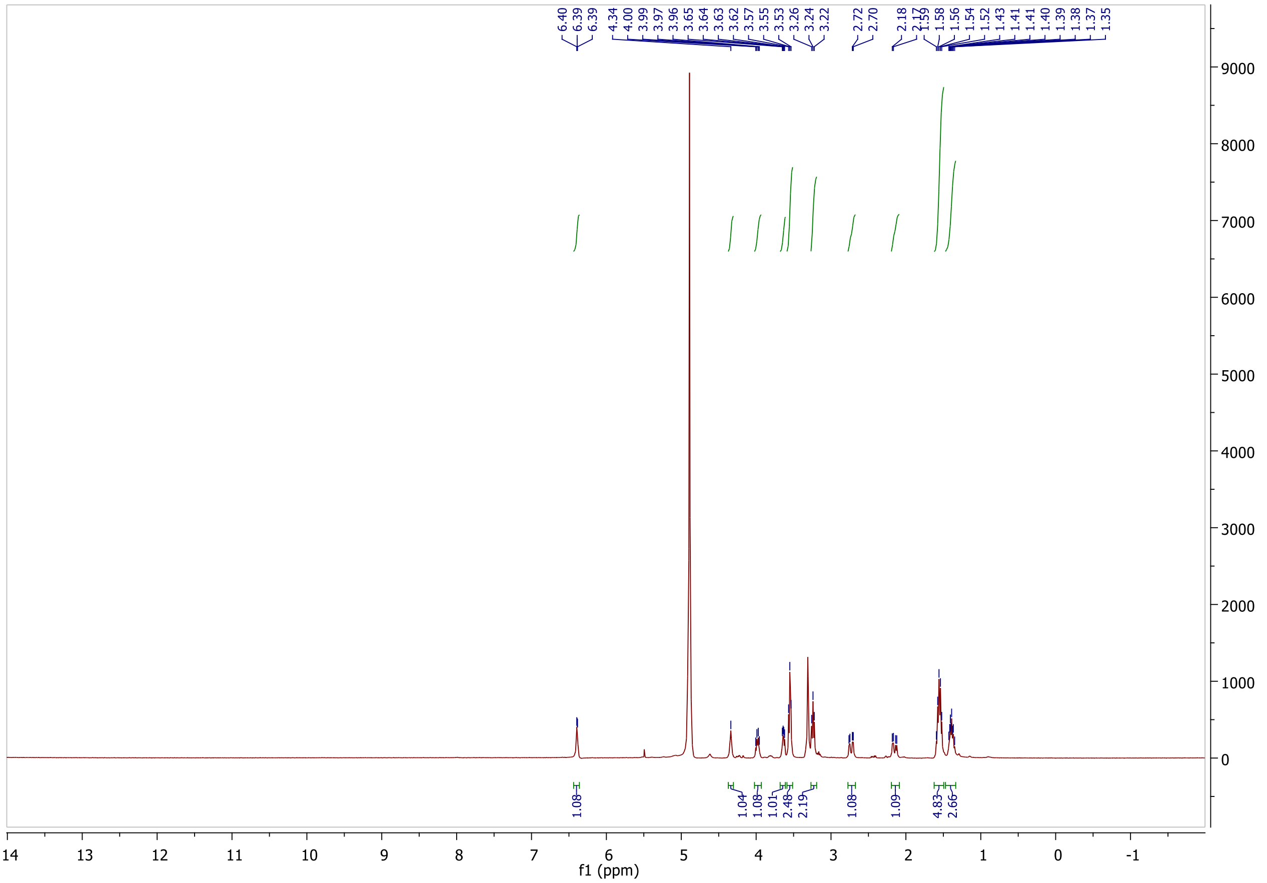

^{13}C-NMR

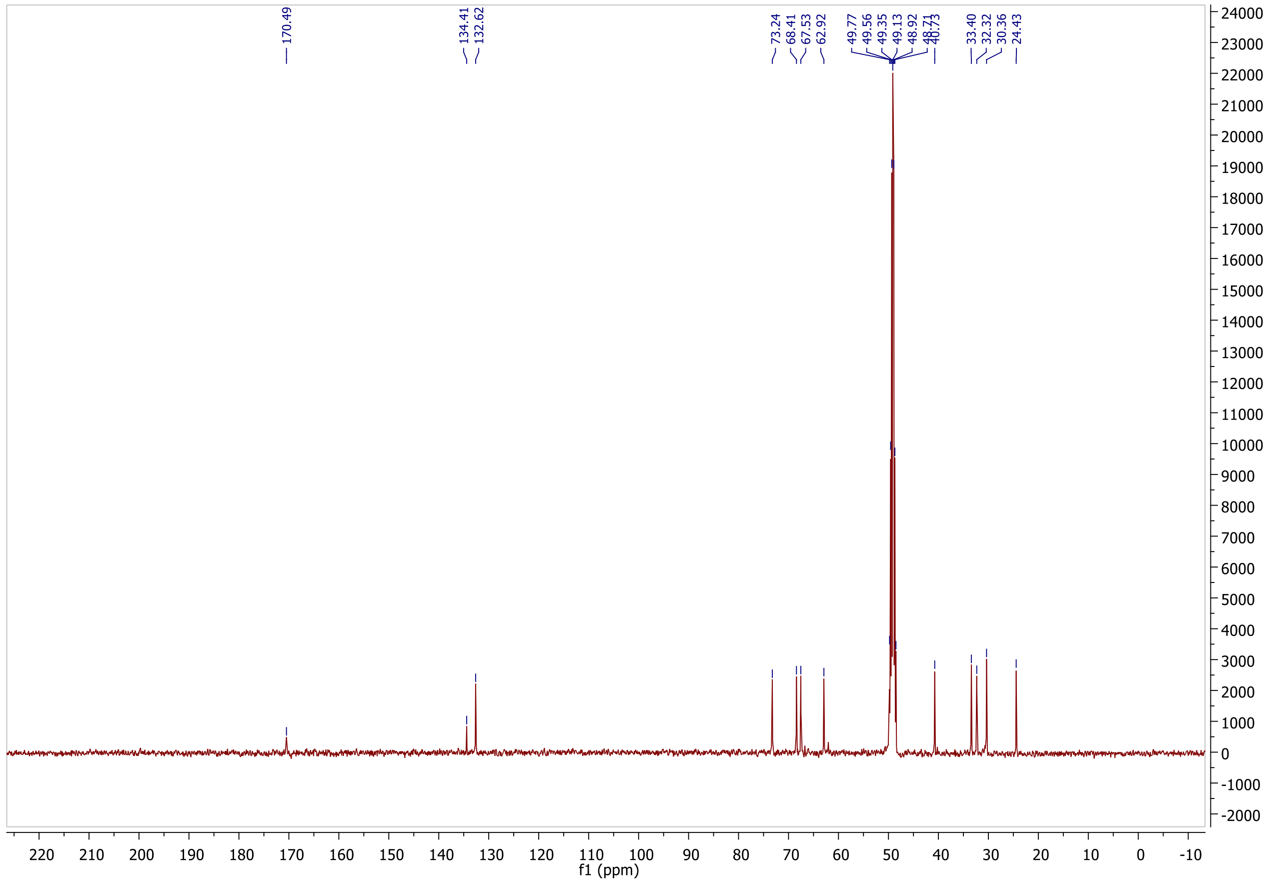

Compound **3d: ^{1}H-NMR**

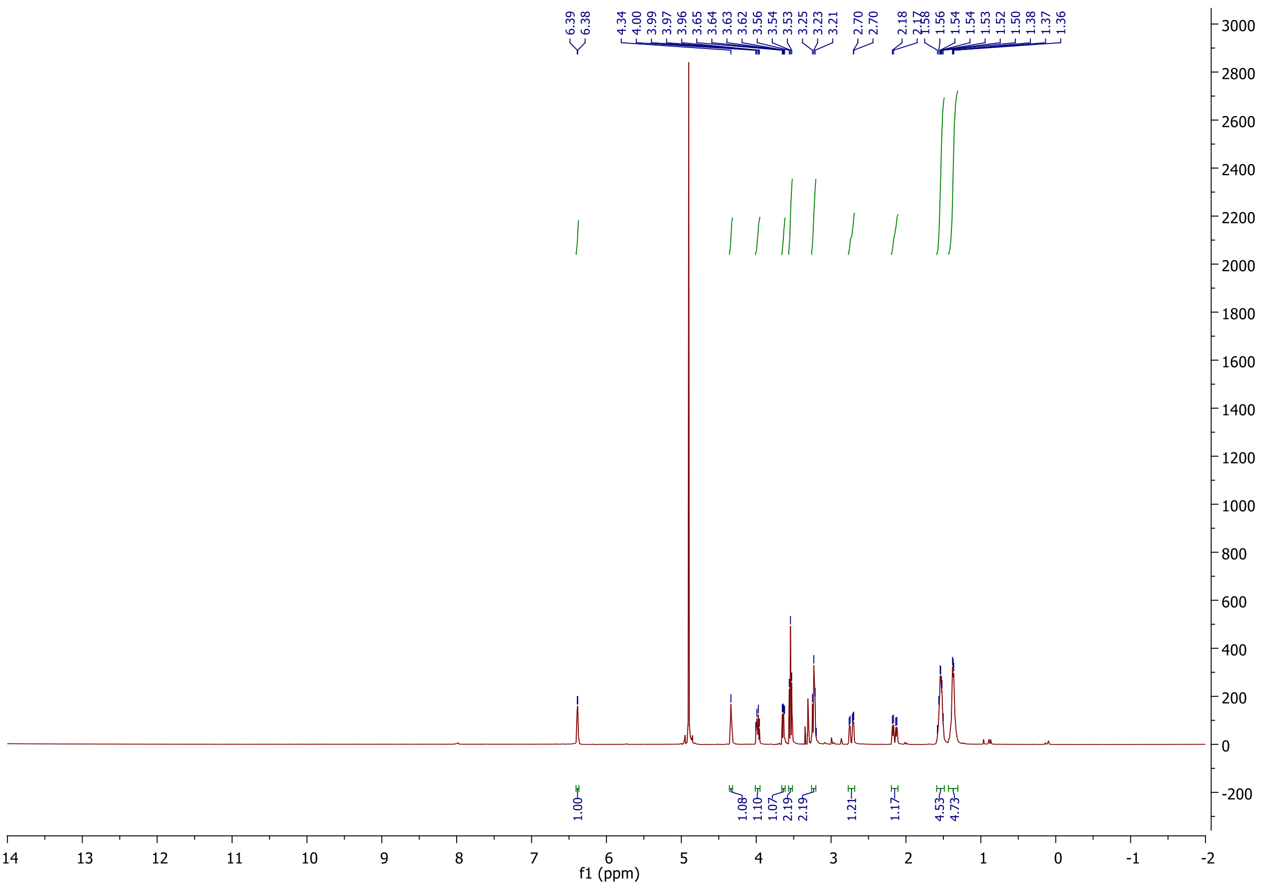

^{13}C-NMR

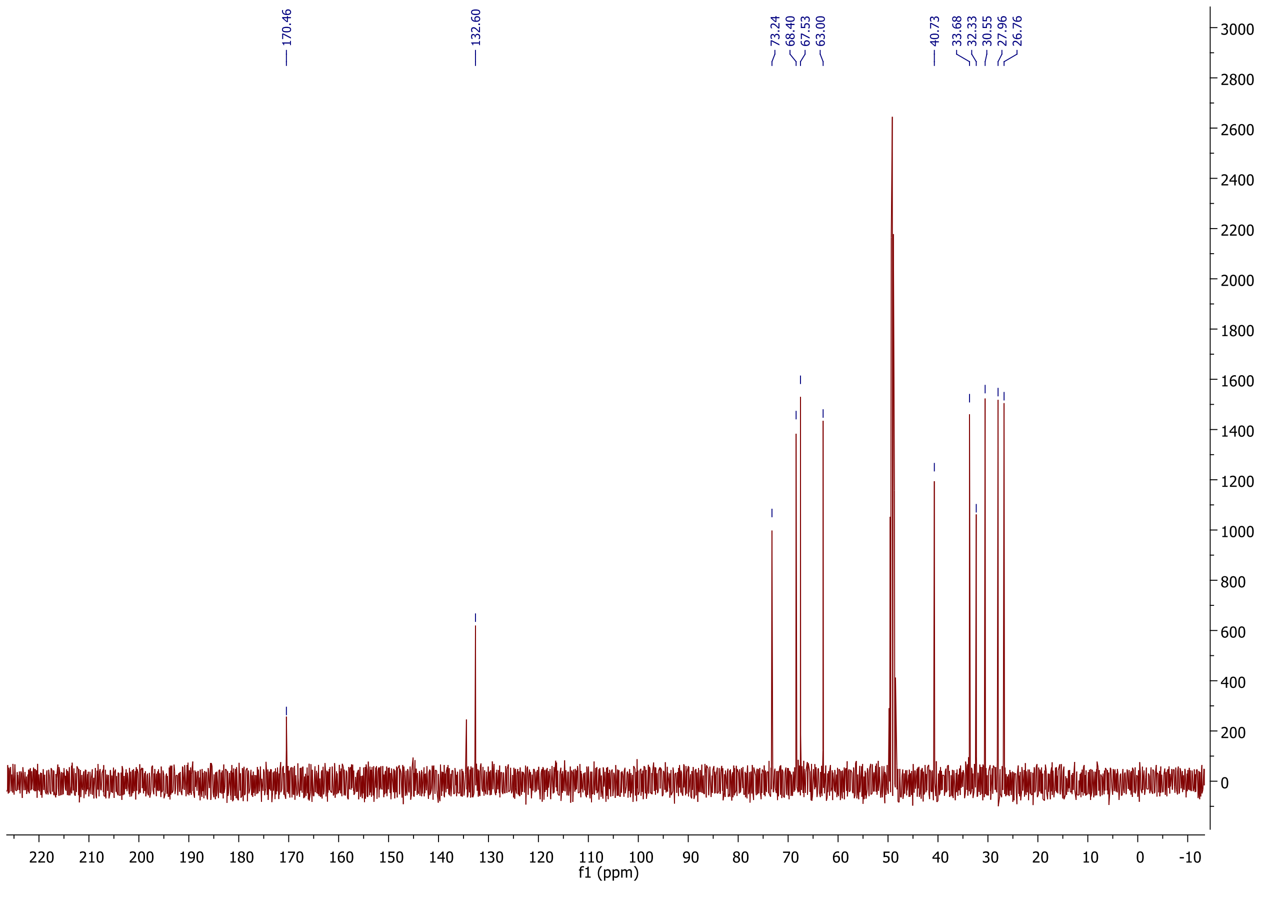

Compound **4a:** ^{1}**H-NMR**

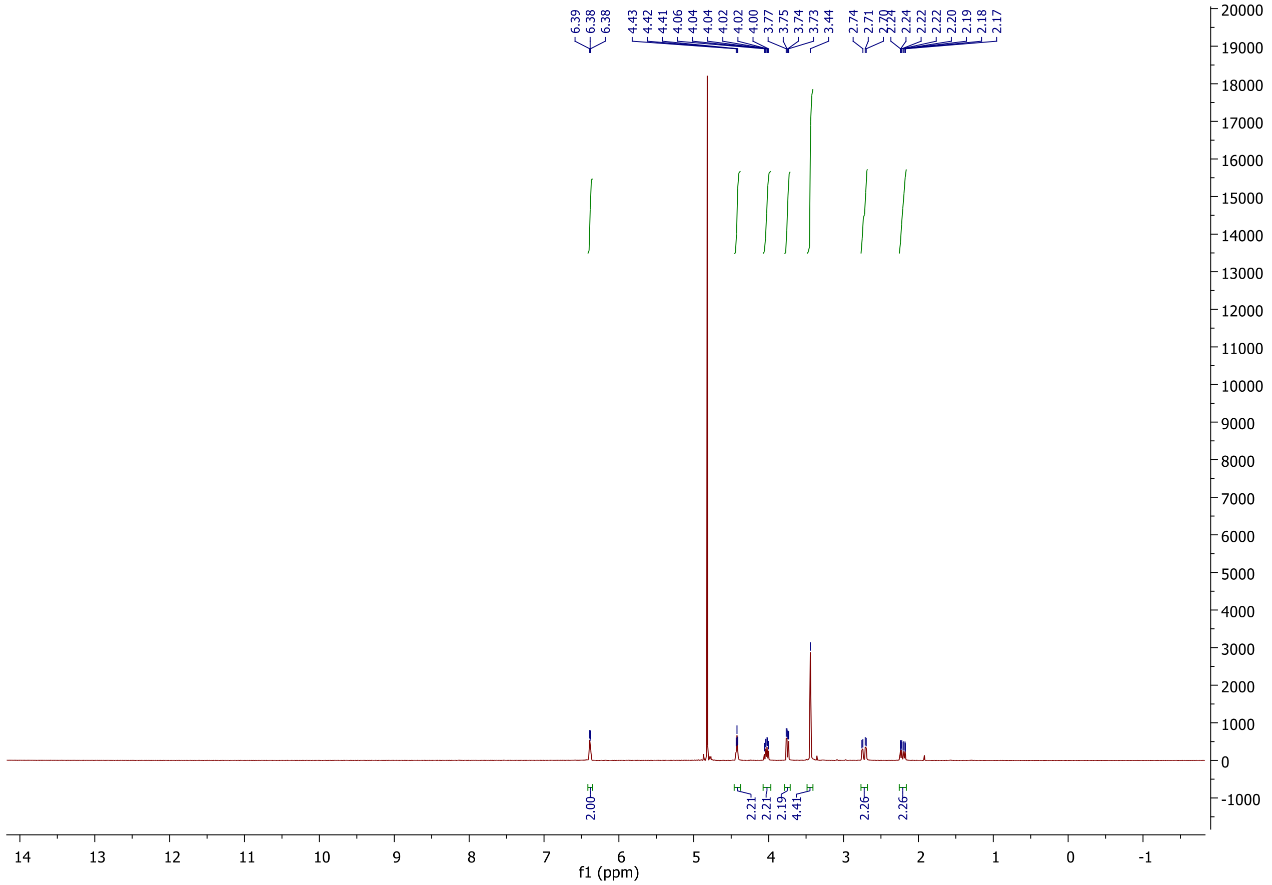

^{13}**C-NMR**

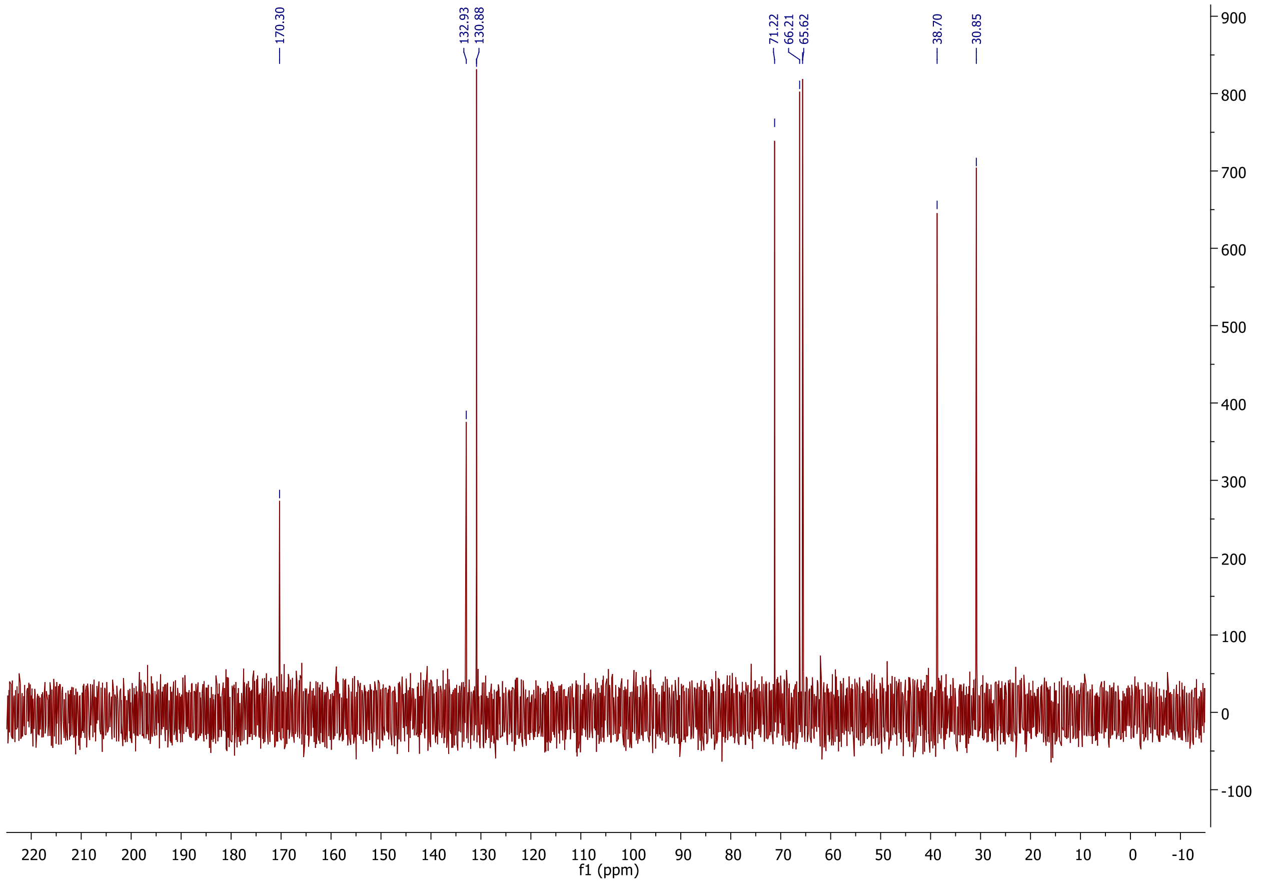

Compound **4b:** ^{1}**H-NMR**

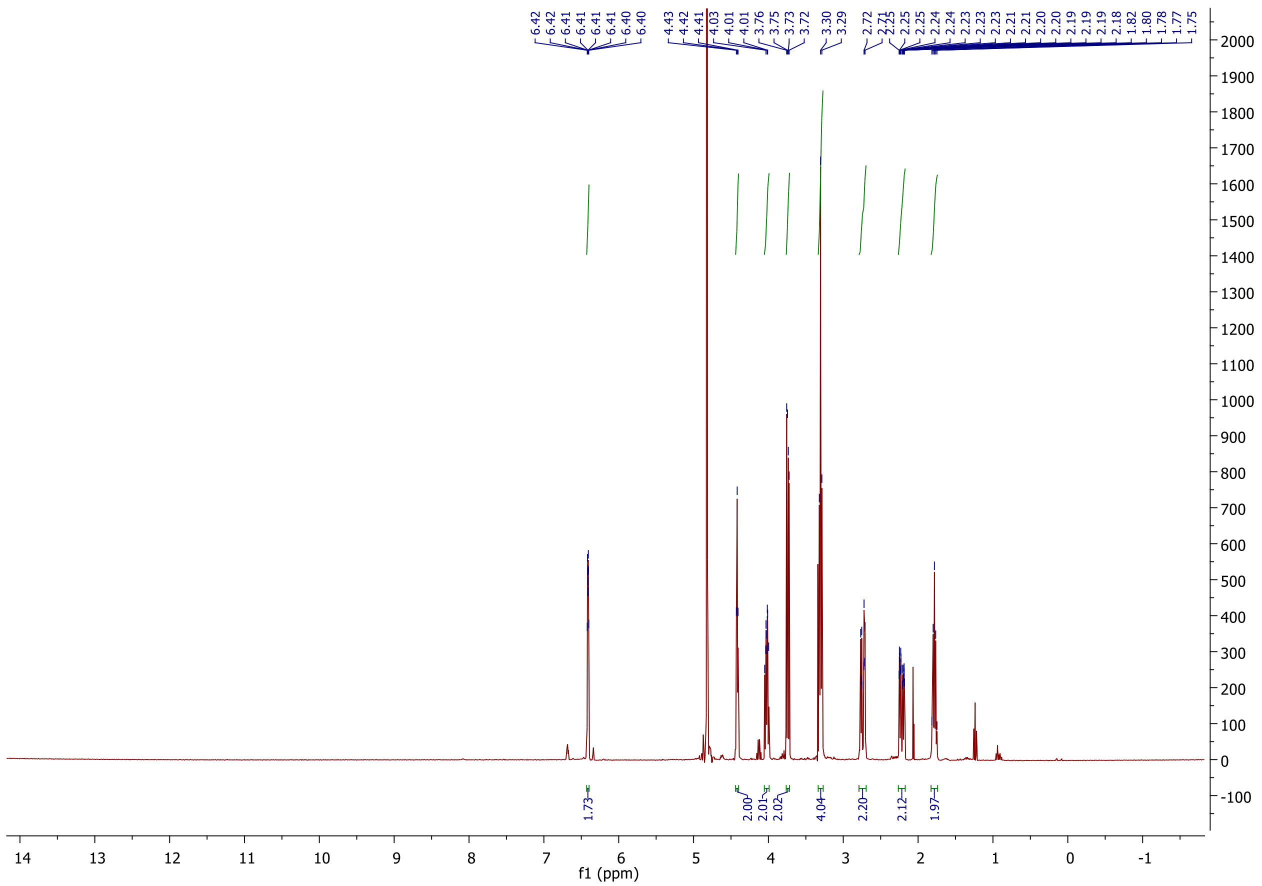

^{13}**C-NMR**

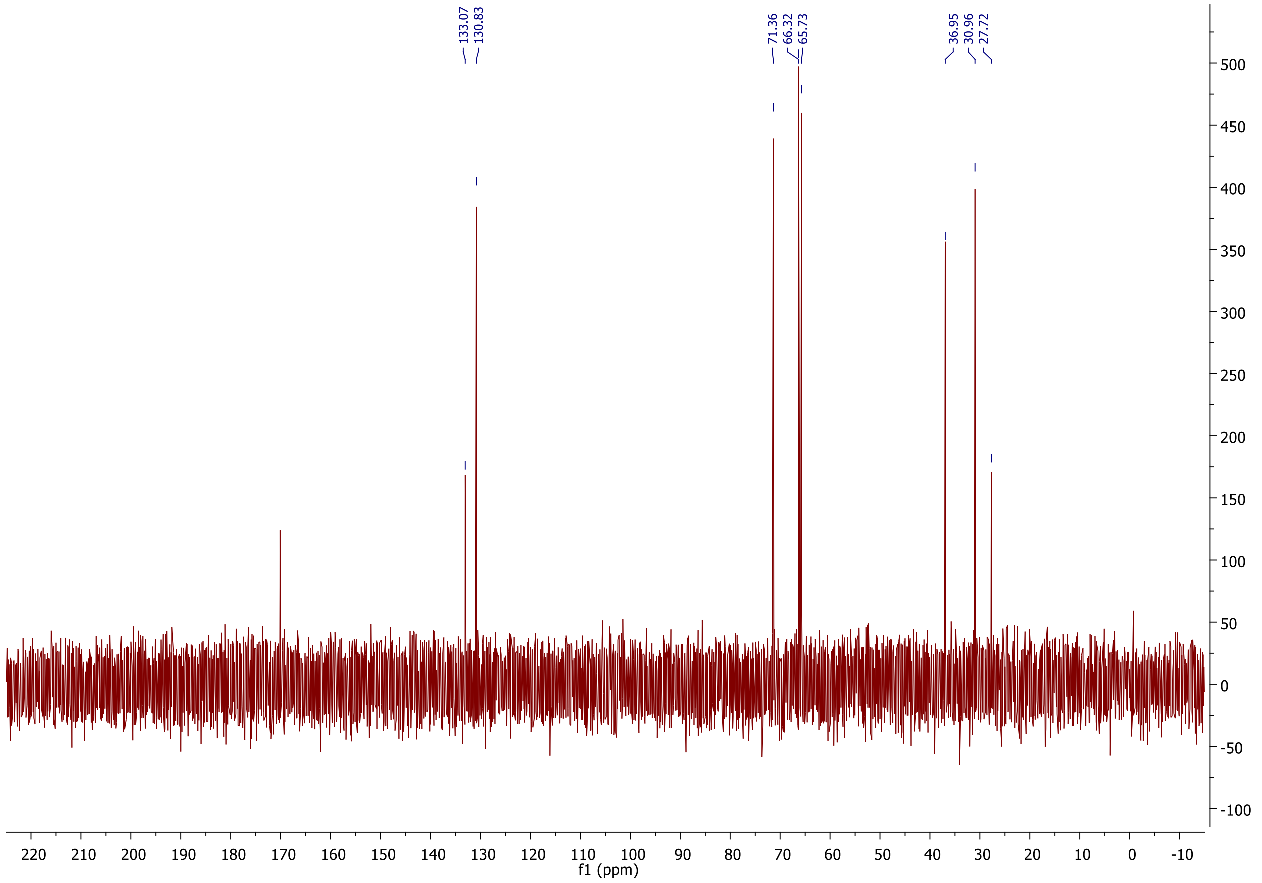

Compound **4c:** ^{1}**H-NMR**

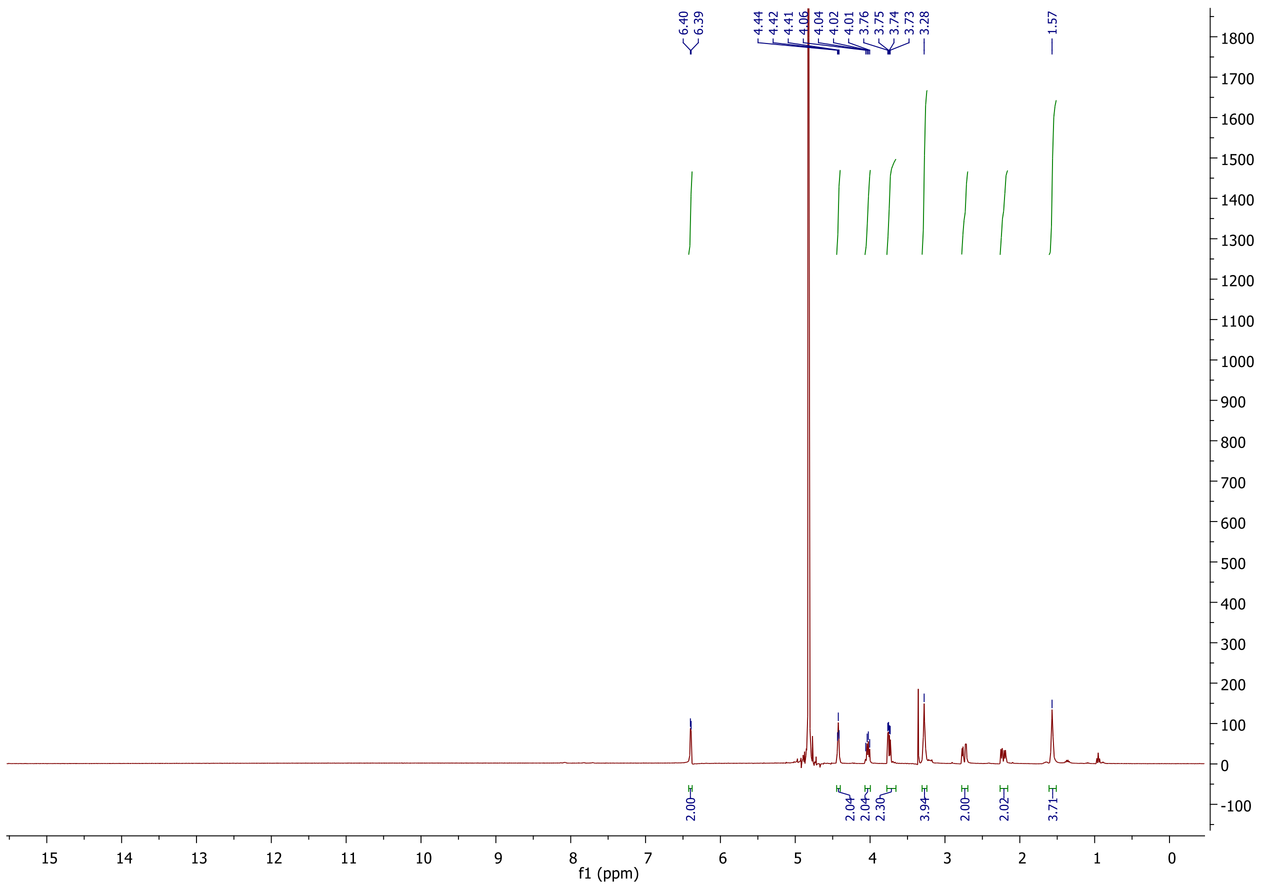

^{13}**C-NMR**

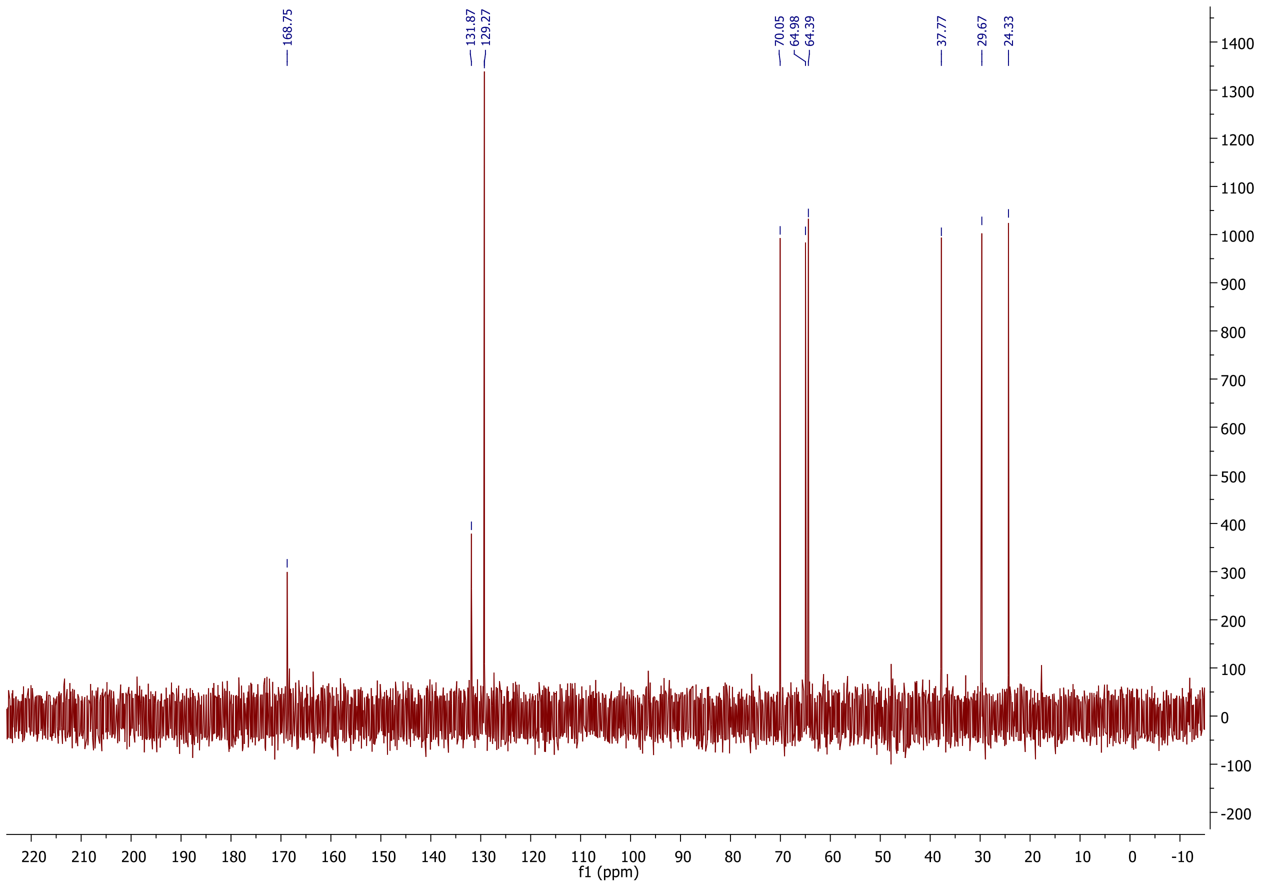

Supplement: IENZ_1422125_Supplementary_Material.pdf [file IENZ_A_1422125_SM8914.pdf]
